# Antioxidants Protect against Arsenic Induced Mitochondrial Cardio-Toxicity

**DOI:** 10.3390/toxics5040038

**Published:** 2017-12-05

**Authors:** Clare Pace, Ruben Dagda, Jeff Angermann

**Affiliations:** 1 Department of Environmental Science and Health, University of Nevada, Reno, NV 89557, USA; clare.pace@gmail.com; 2 Department of Pharmacology, University of Nevada, Reno School of Medicine, Reno, NV 89557, USA; rdagda@med.unr.edu; 3 School of Community Health Sciences, University of Nevada, Reno, NV 89557, USA

**Keywords:** arsenic toxicity, cardiovascular, antioxidant, superoxide, oxidative stress

## Abstract

Arsenic is a potent cardiovascular toxicant associated with numerous biomarkers of cardiovascular diseases in exposed human populations. Arsenic is also a carcinogen, yet arsenic trioxide is used as a therapeutic agent in the treatment of acute promyelotic leukemia (APL). The therapeutic use of arsenic is limited due to its severe cardiovascular side effects. Many of the toxic effects of arsenic are mediated by mitochondrial dysfunction and related to arsenic’s effect on oxidative stress. Therefore, we investigated the effectiveness of antioxidants against arsenic induced cardiovascular dysfunction. A growing body of evidence suggests that antioxidant phytonutrients may ameliorate the toxic effects of arsenic on mitochondria by scavenging free radicals. This review identifies 21 antioxidants that can effectively reverse mitochondrial dysfunction and oxidative stress in cardiovascular cells and tissues. In addition, we propose that antioxidants have the potential to improve the cardiovascular health of millions of people chronically exposed to elevated arsenic concentrations through contaminated water supplies or used to treat certain types of leukemias. Importantly, we identify conceptual gaps in research and development of new mito-protective antioxidants and suggest avenues for future research to improve bioavailability of antioxidants and distribution to target tissues in order reduce arsenic-induced cardiovascular toxicity in a real-world context.

## 1. Introduction

### 1.1. Arsenic and Cardiovascular Disease

Exposure to arsenic through contaminated groundwater is widespread in certain regions of many countries including Bangladesh, India, and China [1]. Arsenic is a potent cardiovascular toxicant; epidemiological evidence has linked arsenic exposure to ischemic heart disease, cerebrovascular disease, atherosclerosis, and hypertension in exposed human populations [2,3,4]. 

Arsenic has been characterized as a strong carcinogen [5]. Arsenic-induced reactive oxygen species (ROS) cause genetic mutations and cancer by promoting DNA damage, activating oncogenic kinases, and activating lipids and proteins that inactivate DNA repair mechanisms [6,7]. Paradoxically, arsenic trioxide has been used as a therapeutic agent in the treatment of acute promyelotic leukemia (APL). APL is a subtype of acute myeloid leukemia (AML) that is genetically characterized by a specific chromosomal translocation that yields the promyelotic leukemia/retinoic acid receptor alpha (PML/RARA) fusion gene—a DNA-binding transcription factor [8]. Arsenic targets the PML moiety of PML/RARA and disrupts PML nuclear bodies, which regulate stem cell self-renewal [8]. Specifically, arsenic was found to bind to cysteine thiols on pyruvate kinase M2 (PKM2), located on the surface of PML/RARA [9]. PKM2 is a glycolytic enzyme that promotes aerobic glycolysis (the “Warburg effect”) leading to tumorigenesis and cancer cell proliferation. Arsenic reduces PKM2 activity, thus inhibiting cancer cell growth [9]. 

Arsenic also prevents cancer cell proliferation in human breast MCF-7 cancer cells by binding to thioredoxin reductase (TrxR) in the thioredoxin (Trx) system [10]. The Trx system, which contains NADPH, TrxR and Trx, is an important thiol-dependent electron donor system in the cell that regulates numerous cell functions including cell viability and proliferation [11,12]. As Trx and TrxR are overexpressed in many aggressive tumors, the Trx system has become an important target for cancer drug development [13,14]. Lu et al. (2007) demonstrated that arsenic trioxide irreversibly inhibits mammalian TrxR by binding at the N-terminal redox-active dithiol and the C-terminal selenothiol-active sites [10]. Despite the benefits, the clinical utility of arsenic trioxide is limited due to arsenic’s severe cardiovascular side effects such as QT-prolongation, ventricular arrhythmias, Torsades de Pointes, and sudden cardiac death [15,16,17].

### 1.2. Mitochondria in Cardiovascular Disease

The cardiovascular toxicity of arsenic is mediated, in part, through mitochondrial dysfunction. Due to their significant energetic requirements, cardiovascular tissues generally have high mitochondrial densities and are thus particularly susceptible to mitochondrial toxins [18]. The electron transport system (ETS) is a critical mitochondrial mechanism. Components of the ETS are particularly susceptible to the toxic effects of arsenic, leading to decreased ATP production, reduced mitochondrial transmembrane potential, and increased ROS [18]. 

### 1.3. Oxidative Stress

ROS cause oxidative modifications of cellular macromolecules including proteins, lipids, and polynucleotides. ROS include superoxide radical (O_2_**•**) which is converted into hydrogen peroxide (H_2_O_2_) spontaneously or via dismutation catalyzed by superoxide dismutase (SOD) [19]. O_2_**•** also reacts with nitric oxide (NO) to generate peroxynitrite (ONOO^−^), an oxidant and reactive nitrogen species. Electron leakage from the mitochondrial electron transport chain (ETC) to molecular oxygen generates a steady stream of O_2_**•**, and other enzymes (NADPH, oxidases, lipoxygenase, cyclooxygenase, cytochrome P450s, and xanthine oxidases (XOs)) also participate in ROS generation [20,21]. 

Intrinsic antioxidants such as SOD, catalase, and glutathione peroxidase (GPx) are the first line of defense against oxidative stress. Excessive ROS or diminished innate antioxidant capacity result in increased oxidative stress and are associated with a multitude of downstream effects as well as disease initiation and progression [22].

### 1.4. Antioxidant Phytonutrients

A growing body of evidence demonstrates the mitochondrial toxicity of arsenic in cardiovascular tissues. Importantly, antioxidant phytonutrient compounds counteract the toxic effects of arsenic on mitochondria [23]. Antioxidant phytonutrients are naturally occurring chemicals in plants such as vegetables, grains, legumes, seeds, fruits, leaves, flowers, and bark. Polyphenols are a major class of phytochemicals that have non-enzymatic antioxidant activities and scavenge free radicals such as O_2_**•**, and H_2_O_2_ [24].

The present review focuses on in vivo and in vitro studies of arsenic on cardiovascular tissues and cells in which the mitochondrial endpoints of membrane potential, ATP production, ROS, respiratory chain activity, and antioxidant content and activity were analyzed. In particular, we reviewed a comprehensive list of antioxidant phytonutrients used to counteract mitochondrial toxic (mito-toxic) effects of arsenic. The antioxidants discussed here may have a therapeutic value for both individuals chronically exposed to arsenic through groundwater sources as well as patients who require arsenic trioxide therapy for APL. Importantly, we suggest future directions for research and clinical interventions applying antioxidant therapy to revert mitochondrial dysfunction, oxidative stress and cell death of cardiovascular tissues in humans exposed to high levels of arsenic trioxide.

### 1.5. Mitochondria

#### 1.5.1. Energy Production

The primary function of mitochondria is to produce energy in the form of ATP through oxidative phosphorylation [25]. Glucose is broken down into pyruvate to generate NADH, which is oxidized in the presence of oxygen (aerobic respiration) or in limited oxygen conditions (anaerobic fermentation) [18]. Pyruvate is actively transported through the inner mitochondrial membrane into the matrix where it is oxidized and combined with coenzyme A to generate CO_2_, acetyl CoA, and NADH [26]. During aerobic respiration, acetyl CoA is then oxidized to generate CO_2_, NADH and FADH_2_, which donate electrons for the electron transport chain, and ATP. NADH and FADH_2_ are also produced via glycolysis in the cytosol and imported to the mitochondrial matrix [18]. Electrons from NADH and FADH_2_ are used in the process of oxidative phosphorylation to donate electrons for the respiratory chain during oxidative phosphorylation [18]. 

The respiratory chain consists of five large protein complexes called NADH-Q oxidoreductase, Q-cytochrome c oxidoreductase, cytochrome c reductase, cytochrome-c-oxidase, and ATP synthase (complexes I, II, III, IV, and V) [18]. The transfer of electrons sets up an electron gradient used to pump protons across the inner membrane space. Protons move down their concentration gradient back to the matrix, where they combine with O_2_ to form H_2_O and serve to power ATP synthase (Complex V) to facilitate the generation of ATP from ADP and inorganic phosphate [25]. 

Despite the overall efficiency of the respiratory chain, a small amount of electron leakage occurs at mitochondrial complexes I and III. This charge leakage can reduce oxygen to form reactive oxygen species such as O_2_, H_2_O_2_, and ^−^OH [18]. Deficiencies in mitochondrial energy production can be demonstrated in an experimental setting through decreased ATP content, decreased activity of mitochondrial complexes, and elevated ROS, particularly mitochondrial superoxide, suggesting decreased respiratory chain efficiency [18]. 

#### 1.5.2. Calcium Storage

In addition to other intracellular storage deposits, calcium is transiently stored in the mitochondria and used for signal transduction and regulation of intracellular reactions. Calcium is taken up into the mitochondrial matrix by the calcium uniporter in the inner mitochondrial membrane, which is driven by mitochondrial membrane potential [27]. Calcium can later be released via a sodium-calcium exchange protein or by a change in the membrane potential [27]. Dysfunction in mitochondrial calcium status, such as Ca^2+^ overload, can result in elevated oxidative stress through Ca^2+^-stimulated NO production, Ca^2+^-induced cardiolipin peroxidation, Ca^2+^-induced mitochondrial permeability pore opening with consequent release of cytochrome c and GSH-antioxidative enzymes, and activation of Ca^2+^-calmodulin dependent protein kinases [27]. Additionally, the rise in mitochondrial ROS can alter Ca^2+^ dynamics and increase Ca^2+^ surge. Thus the reciprocal interaction between Ca^2+^ induced ROS increase and ROS-modulated Ca^2+^ upsurge can create a self-amplified loop [27]. 

#### 1.5.3. Apoptosis

Mitochondrial-mediated apoptosis is an important mechanism of programed cell death. Pro-apoptotic proteins on the surface of mitochondria detect mitochondrial damage and activate BAX proteins, which “punch” pores in the outer mitochondrial membrane, allowing cytochrome c to be released into the cytoplasm, where it binds to apoptotic protease activating factor-1 (Apaf-1) [26,28]. The interaction of these proteins forms apoptosomes, which bind and activate caspase-9, and subsequently cleave mitochondrial membrane proteins and result in phagocytosis [26,28]. Elevated levels of BAX, Apaf-1, and caspases 3, 8, and 9 all suggest an increase in apoptosis. Other biomarkers of apoptosis include upregulation of TGF-β, a profibrogenic cytokine that indicates repressed cell proliferation and apoptosis, and phosphorylation of the NF-κB pathway.

#### 1.5.4. Membrane Potential

Mitochondria closely regulate membrane potential, which is critical for mitochondrial functions including the electron transport chain and calcium homeostasis [18]. A significant loss of membrane potential depletes the cell of ATP and results in cell death [29]. Dysfunctional membrane potential can be measured fluorescently by the uptake of ratiometic mitochondrial membrane specific dye JC-1, and by measuring the membrane pore permeability and Na^+^/K-ATPase activity. 

Protein kinases are translocated to mitochondria in response to oxidative stress, where they regulate membrane potential, impact growth factors, and lead to phosphorylation of respiratory chain proteins. The mitogen-activated protein kinase (MAPK) cascade includes the subfamilies p38 mitogen-activated protein kinase (p38 MAPK), c-Jun N-terminal kinase (JNK), and extracellular signal-regulated kinase (ERK). p38 MAPK signaling regulates cell death, in part, by initiating the translocation of BAX from the cytosolic to mitochondrial compartments [29]. Activated JNK stimulates apoptosis by inhibiting the anti-apoptotic Bcl-2 and Bcl-xl, leading to cytochrome c release and subsequent induction of apoptosis. Increased ERK1/2 activity leads to ERK1/2 colocalization to the mitochondria where it promotes autophagy and mitochondrial degradation [30]. This creates a self-amplifying loop of mitochondrial dysfunction in which mitochondrially-derived ROS activates ERK1/2, which translocates to the mitochondria where it damages ATP synthase function, reduces mitochondrial membrane potential, and causes cytochrome c release, leading to further ROS release [29].

#### 1.5.5. Source of ROS

Mitochondria are an important source of ROS, which play a significant role in cell signaling [18]. As previously mentioned, some ROS are produced as a result of electron leak that occurs due to the incomplete flow of electrons in complex I and in complex III. ROS levels are typically low and are maintained in balance through the activity of intrinsic antioxidants, which scavenge for ROS. For example, superoxide dismutases (SODs) convert O_2_^−^ to H_2_O_2,_ and catalase subsequently converts H_2_O_2_ to water. Another endogenous antioxidant is glutathione reductase (GSH), which donates a reducing equivalent (H^+^ + e^−^) to neutralize ROS. By donating an electron, GSH becomes reactive and binds to another reactive GSH to form glutathione disulfide (GSSG). Oxidized glutathione can be converted back to its reduced state using NADPH as an electron donor, and the ratio of GSH/GSSG is often used as a measure of oxidative stress [31]. Glutathione S-transferase (GST) utilizes GSH as a substrate and participates in the metabolism of xenobiotics [32], and glutathione peroxidase (GPx) and thioredoxin reductase (TR) catalyze interconversion and equilibrium among reduced/oxidized species [21]. 

Excessive ROS or decreased content/activity of endogenous antioxidants result in oxidative damage to cells and phospholipid membranes [25]. Studies have used a variety of methods to assess oxidative stress. A direct measure of ROS can be obtained by measuring the fluorescence of 2′,7′-dichlorofluorescin diacetate (DCFH-DA) to 2′,7′-dichlorofluorescein (DFA) [33]. DCFH-DA readily diffuses into cells, is hydrolyzed to H_2_ DCF (which is not membrane permeable), and then is oxidized by H_2_O_2_ and other ROS to the fluorescent compound DFA. The fluorescence of DFA is proportional to the intracellular concentration of combined H_2_O_2_, ONOO^−^, and OH^−^ [34].

#### 1.5.6. Arsenic and Cysteine Thiol Binding

Arsenic is considered a “sulfhydryl-reactive metalloid” [35]. The affinity of arsenic for free sulfhydryl groups results in certain mechanisms of toxicity, including binding and depletion of GSH pools, as well as binding and inactivation of sulfhydryl-rich proteins [32]. This cysteine thiol binding-mediated inactivation of specific proteins is central to the ability of arsenic to both induce metallothionein biosynthesis [36] and elevate ROS generation via inhibition of the antioxidant enzyme thioredoxin reductase [10]. The depletion of GSH titers by arsenic may also influence arsenic methylation rates, leading to variable rates of metabolic activation in certain tissues [37]. Protein cysteine thiol binding in particular tissues, including cardiac muscle, results in decreased proteinaceous antioxidant activity, leading to accumulation of ROS and attendant pathological consequences [38,39,40,41]. 

#### 1.5.7. ROS and the Nrf2 Pathway

Arsenic’s affinity for sulfhydryls also contributes to the activation of Nuclear factor erythroid 2-related factor (Nrf2) [33]. Kelch ECH (Keap-1) anchors Nrf2 in the cytoplasm in quiescent conditions. Arsenic binds to Keap-1 cysteine residues, leading to Nrf2 dissociation and translocation to the nucleus [42,43]. Nrf2 activation results in a coordinated antioxidant and anti-inflammatory response in which phase II detoxification enzymes are activated. Nrf2 regulates GPx, GST, SOD, and TR (all discussed above) as well as glucose-6-phosphate (G6PD)-which provides NADPH to glutathione reductase, heme oxygenase-1 (HO-1)- which generates antioxidant molecules and regulates apoptosis, glutathione reductase (GR)- which catalyzes the reduction of GSSG to GSH, and NAD(P)H:quinone dehydrogenase (NQO1)- a FAD-binding protein [42]. 

#### 1.5.8. Arsenic-Induced Mitochondrial Toxicity

Arsenic, a recognized cardiovascular toxicant, has exhibited mito-toxic effects in vascular smooth muscle [44], myocardial cells [45], and vascular endothelial cells [46]. Arsenic is associated with mitochondrial dysfunction through mechanisms such as elevated ROS, induction of apoptosis, and Ca^2+^ overload. In the heart, arsenic causes QT prolongation by altering L-type calcium channels [47]. As previously noted, GSH is also consumed during metabolism of arsenic and in response to arsenic-induced ROS. Indeed, GST utilizes GSH as a substrate and participates in the xenobiotic metabolism of arsenic and other compounds [32] and GPx catalyzes the reduction of H_2_O_2_ to H_2_O with GSH as a substrate [32,48]. Furthermore, decreased availability of GSH due to arsenic toxicity further contributes to ROS accumulation [49]. 

The pathological effects of ROS are widespread for the cell. For example, lipid peroxidation is a common feature of oxidative stress that has also been attributed to arsenic exposure. In this process, free radicals cause cell damage by sequestering electrons from the lipids in cell membranes [50]. Additionally, arsenic may decrease membrane-based enzyme activity through the generation of ROS [32]. For instance, ROS inhibit the activity of Na^+^/K^+^ ATPase resting membrane homeostasis, which is critical for maintaining mitochondrial transmembrane potential [51]. 

In light of arsenic’s demonstrated cardiovascular toxicity and the association between mitochondrial dysfunction and ROS, the present study explores mechanisms of arsenic toxicity and the protective action of food-based antioxidants, many of which work by scavenging antioxidants. We hypothesize that studies provide sufficient proof-of-concept that antioxidant formulations can protect against arsenic-induced mitochondrial dysfunction in cardiovascular cells and tissues.

## 2. Methods

We conducted a literature search by screening MEDLINE, Web of Science, and Google Scholar for the terms “mitochondria” “mitochondrial” “toxicity” “toxic” in combination with “heart” “cardiac” “cardiovascular” “myocardial” “myocardium” “arsenic” “ATO” or “arsenic trioxide” and “antioxidant”. We limited our results by eliminating reviews and publications that were not available in English. We had two primary objectives. The first was to test the hypothesis that non-enzymatic antioxidants are highly effective against arsenic-induced cardiovascular toxicity, and may be employed to protect mitochondria in cardiovascular cells and tissues. The second objective of this review was to identify a series of food-based antioxidants that could be supplemented to the diet of arsenic exposed individuals to improve cardiovascular heath, and/or developed into commercial formulations to include in clinical trials involving arsenic trioxide.

**Figure 1:**
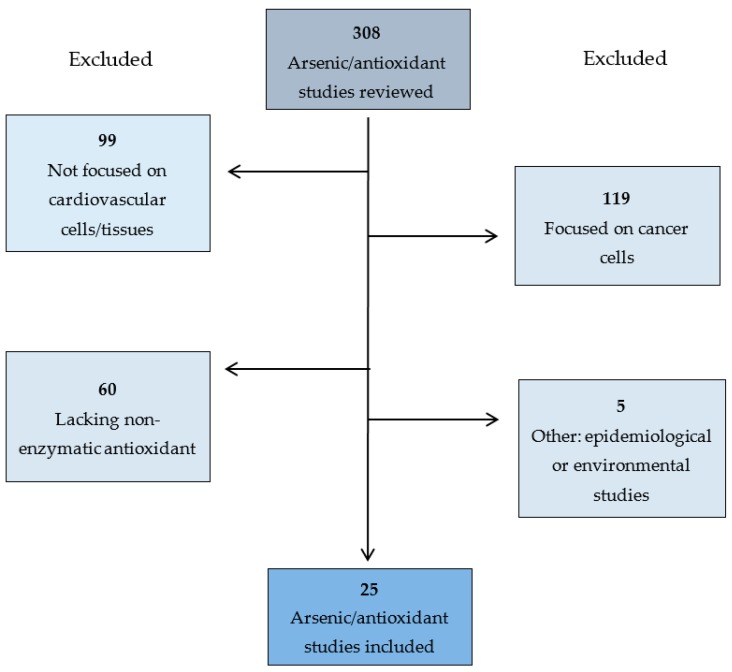
Schematic of selected references.

Appropriate hits were analyzed in detail and reference lists were screened for additional appropriate studies. Our initial search returned 308 potential studies to include. One hundred and nineteen studies were eliminated because they were focused on the effects of arsenic on cancer cells, 99 focused on non-cardiovascular cell or tissue types (10 blood, 37 liver/kidney, 16 brain and nervous system, 8 reproductive system, and 28 other), 60 did not investigate the restorative effects of a non-enzymatic antioxidant, 3 had an environmental focus, and 2 were epidemiological studies (Figure 1). The remaining 25 articles were included in the present review. We assessed the quality of the studies analyzed in this review in terms of clarity of purpose, experimental design, appropriateness of statistical methods used, thoroughness of data reporting, and impact of primary research article and journal in which the article was published. No additional studies were collected from Web of Science or Google Scholar. We included animal model investigations and cell culture studies in this review. Our search yielded studies investigating isolated heart mitochondria, cardiomyocytes, and cardiovascular tissues.

## 3. Results

### 3.1. Arsenic

#### 3.1.1. Dose and Duration

**Figure 2:**
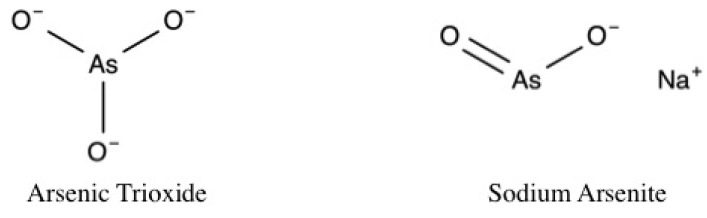
Arsenic species.

Dose selection justifications varied between studies, but generally, in vivo doses were selected based on the literature [33,52,53,54,55]. For example, as a fraction of the lethal dose [56], or experimentally as the minimum dose that induced cardio-toxic effects [57]. In vitro arsenic doses were selected based on significant detrimental effects on cell viability and/or cell death and were further stratified by exposure duration (Table 1). The studies presented in this review utilized arsenic trioxide (As_2_O_3_) [32,45,53,54,57,58,59,60,61,62,63,64,65,66] or sodium arsenite (NaAsO_2_) (Figure 2) [49,55,56,67,68,69,70,71] at in vivo doses ranging from 0.8 mg/kg/day to 5 mg/kg/day administered intravenously or 2 mg/kg/day to 50 mg/kg/day administered orally for durations of 6 to 56 days. Experimental animals included Sprague Dawley rats [49,55,70], Wistar rats [59,67], BABL/c mice [72], APO E-/- mice [71], and guinea pigs [58]. In vitro exposures ranged from 1 μM to 20 μM arsenic at exposure durations of <3 to 72 h. Cell types included neonatal ventricular cardiomyocytes (NRLVM) [64], rat embryonic cardiomyocytes (H9c2) [62], primary cardiomyocytes isolated from Albino Wistar rats [69], and primary guinea pig cardiomyocytes [58]. Although not the focus of the present review, one study also utilized human promyelotic leukemia (NB4) cells [65]. 

#### 3.1.2. Cardiovascular Structure and Function

In vivoarsenic exposure increased arsenic deposition in the heart [53], caused QT interval prolongation [70], increased QTc interval [54], induced ST-T wave change [62], prolonged PQ interval [59], increased RR and QRS interval [70], inhibited I_KS_ current [58], reduced I_K_ amperage [58] decreased heart rate [70], decreased cardiac output [64], and decreased 3′-5′-cyclic adenosine monophosphate (cAMP), an intracellular messenger that mediates catecholaminergic control of heart rate and contractility [74]. Arsenic exposure increased triglycerides [55], total cholesterol [67], and LDL cholesterol [70], and decreased HDL cholesterol in blood plasma [68]. Arsenic decreased phospholipids, increased free fatty acids (FFA) [56], and increased lipase activity in heart tissues [63]. Arsenic also increased atherosclerotic plaque formation in the aortic arch and aortic sinus [71], and decreased atherosclerotic plaque stability by increasing macrophage content [71].

Arsenic significantly elevated Creatine Kinase (CK) [62] and Creatine Phosphokinase (CPK) [68], which indicate stress to the heart, and elevated Creatine Kinase from muscle and brain (CK-MB) [70], as well as levels of serum troponin [54], which are cardiac markers used to diagnose acute myocardial infarction. Arsenic increased inflammation, measured with C-reactive protein (CRP) [63], and increased the activities of cardiac enzymes lactate dehydrogenase (LDH) [55,70], aminotransferase (AST) [56], alkaline phosphatase (ALP) [56,70], and aspartate aminotransferase (ALT) [56,70], all of which indicate necrotic damage to the heart [75]. Furthermore, arsenic caused notable changes in cardiac structures including cytoplasmic vacuolization, myofibrillar loss, and cardiomyocyte necrosis [72].

#### 3.1.3. Cardiac Nrf2

Several in vivo studies reported downregulation of cardiac Nrf2 following arsenic exposures ranging from 8 to 28 days [70], as well as upregulation of Keap-1 [56]. However, Hu et al. (2016) reported an increase in Nrf2 activity after exposing H9c2 cardiomyocytes to 2 μM/mL arsenic for 24-h. Based on arsenic’s affinity for sulfhydryls, we expected to see arsenic-induced Nrf2 activation, as reported by past studies on other cell types [76,77,78,79,80]. These apparently contradictory results suggest a short term upregulation of the Nrf2 pathway in which Nrf2 is modestly activated because cardiomyocytes have a weak ability to excrete arsenic into the extracellular space [81]. This is presumably followed by a more permanent repression of Nrf2 [81]. 

Since Nrf2 activation stimulates expression of phase II detoxification enzymes, Nrf2 repression should decrease the innate antioxidant response. Indeed, arsenic decreased the activity of enzymes in the Nrf2 pathway including SOD [70], TR [45], GPx [67], GR [56], GST [67], G6PD [56], and hemeoxygenase 1 (HO-1) [70]. Again Hu et al. (2016) had contradictory findings, reporting elevated HO-1 expression, and an increase in NQO1 that likely resulted from short-term elevation in Nrf2. 

Arsenic exposure decreased catalase activity [67], decreased GSH levels [68], and decreased GSSG levels and/or GSH/GSSG ratio [67]. In one study, GSH level was elevated at 6 h following 5 μM arsenic exposure [66], but this trend reversed at 12 and 24 h [66], a result that is consistent with other studies. This observation suggests that arsenic may induce a short-term, compensatory but unsustainable increase in GSH activity. As previously mentioned, GSH is used in the biotransformation of inorganic arsenic to methylated arsenic species, and a temporary increase in GSH may suggest a short-term effect related to arsenic metabolism [66]. 

#### 3.1.4. ROS

In addition to measuring antioxidant activity, many studies have reported ROS levels induced by arsenic exposure. Indeed, cells exposed to arsenic demonstrated an elevation of several ROS and oxidants including H_2_O_2_ [60,63], mitochondrial superoxide [49,60], ONOO-, and OH- [70]. Arsenic increased lipid peroxidation- measured as malondialdehyde (MDA) [32]- and increased thiobarbituric acid reactive substances (TBARS), a byproduct of lipid peroxidation [67]. Arsenic also increased xanthine oxidase (XO) [66] and increased NOX enzyme activity- an important source of ROS [56]. Interestingly, one study detected a significant increase in nitric oxide (NO) following exposing rats to 5 mg/kg/day sodium arsenite for 28 days [70], and one study found that NO changes were not significant following 3 mg/kg arsenic exposure in mice on 4 alternate days over an 8-day period [33]. NO is a second messenger involved in the process of blood vessel vasorelaxation, and decreased NO in endothelial cells is associated with elevated blood pressure. Paradoxically, low dose arsenic exposure has been shown to increase NO in endothelial cells, whereas higher dose arsenic exposure decreases NO [82]. Thus, reports of increased NO in response to arsenic exposure likely result from arsenic’s non-linear effects based on exposure dose and duration. 

#### 3.1.5. Apoptosis

As expected, arsenic elevated lactate dehydrogenase (LDH) release at various doses and exposure times [33,49,72] and consistently decreased cell viability [60]. Apoptosis was detected via DCFH-DA fluorescence, flow cytometry profile, physiological characteristics (shrinkage, blebbing and rounding up), DNA fragmentation, and/or increased micronuclei frequency [70]. Arsenic also decreased cell growth via DNA synthesis [60]. 

Arsenic increased caspase-3 cleavage [49], activated caspase-3 [60], caspase-8 [61], and caspase-9 [62], and increased cytochrome c levels in the cytosol [69]. Arsenic increased the level of proteolysis of poly (ADP-ribose) polymerase (PARP) (downstream of caspase-3 activation) [69], increased expression of pro-apoptotic proteins BAX [60], Bad [69], and p53 upregulated modulator of apoptosis (PUMA) [60], and decreased expression of anti-apoptotic proteins Bcl-2, Bcl-xL [60], Survivin [60], X-linked inhibitor of apoptosis protein (XIAP) [60], cellular inhibitor of apoptosis protein-1 (CIAP1), and the ratio of phosphorylated to total anti-apoptotic kinase (AKT) [62]. Arsenic also decreased Bcl-2/BAX protein ratio in one study [62]. Arsenic up regulated TGF-β and Smad3 [70], which both indicate apoptosis (TGF-β triggers Smad3 activation). Additionally, arsenic exposure caused the phosphorylation of the nuclear factor-kappa-light-chain-enhancer of activated B cells (NF-κB) pathway [69]. When phosphorylated, NF-κB translocates to the nucleus and induces the transcription of anti apoptotic proteins such as XIAP1 and Survivin, thus, decreased NF-κB activity has a pro-apoptotic effect. Interestingly, one study reported decreased activity of NF-κB, which is generally considered an anti-apoptotic event [60]. This contradiction may stem from the fact that NF-κB has both inflammatory and anti-inflammatory properties, and apoptosis is an essential mechanism that prevents prolonged inflammation. Thus, increased and decreased NF-κB may reflect different states of cellular stress [83]. 

It is also worth noting that although a majority of studies have reported on arsenic’s ability to induce apoptosis in various cell types including aortic, coronary, and mesenteric smooth muscle cells [84,85], several past studies have also reported that exposure to inorganic arsenic results in necrosis whereas the metabolite monomethylarsonous acid (MMA) primarily results in caspase-dependent apoptosis in vascular smooth muscle cells [44] and other cell types [86,87,88]. Variations in the method of cell death may result from differences in the arsenic dose, duration, and cell type (vascular smooth muscle vs. cardiomyocytes). In the present review, we report that among cardiomyocytes, inorganic arsenic promotes ROS mediated apoptosis. 

#### 3.1.6. Calcium Overload

Arsenic increased Ca^2+^ content [32], increased Cav 1.2 protein expression [64], and decreased Ca-ATPase [32]. Calcium overload results in collapsed mitochondrial transmembrane potential. Indeed, arsenic consistently decreased mitochondrial transmembrane potential [64], a sign of decreased mitochondrial health. Arsenic also decreased Na^+^/K^+^ ATPase activity, which regulates mitochondrial transmembrane potential [32,56], and elevated phosphorylation of JNK and p-38 MAPK, which mediate mitochondrial membrane collapse [64].

#### 3.1.7. Mitochondrial Function

Arsenic decreased the activity of mitochondrial complexes I [45], II [70], III [45], and IV [45], decreased steady-state levels of ATP [45], decreased Mg^2+^ATPase, [56], and increased the level of mitochondrial superoxide [60]. Arsenic caused mitochondrial swelling, mitochondrial pore opening [45], and other alterations in mitochondrial morphology [70]. In one study, arsenic decreased the activities of heart mitochondrial enzymes including isocitrate dehydrogenase (ICDH), succinate dehydrogenase (SDH), malate dehydrogenase (MDH), and NADH dehydrogenase [56]. Arsenic also decreased the activity of aconitase, an indicator of increased mitochondrial superoxide and essential enzyme in the citric acid cycle that converts citrate into iso-citrate [89]. Arsenic increased autophagy in cardiomyocytes, as measured by assaying for the level of the autophagic markers microtubule-associated protein 1A/1B-light chain 3-phosphatidylethanolamine conjugate (LC3-II)/microtubule-associated protein 1A/1B-light chain 3 (LC3-I) [65]. In the process of autophagy, LC3 is conjugated with phosphatidylethanolamine to form LC3-phosphatidylethanolamine conjugate (LC3-II), which is recruited to autophagosomal membranes in autophagic vacuoles. The autophagosomes subsequently fuse with lysosomes to form autolysosomes, thus contributing to autophagic cell death [90].

This data is consistent with previous studies showing that arsenic induces widespread mitochondrial dysfunction via loss of mitochondrial membrane potential, generation of ROS, diminution of cytochrome-C oxidase function, ROS-dependent activation of autophagy, and suppression of oxygen consumption via depletion of mtDNA copy number [91,92]. In addition, a recent study found that inorganic arsenic and/or methylated arsenicals decrease ATP content, increase the level of hydrogen peroxide and mitochondrial superoxide, cause aberrant nuclear clustering of mitochondria and decrease mitochondrial content in vascular smooth muscle cells [44].

The studies reviewed here clearly demonstrate that arsenic affects all aspects of mitochondrial function including energy production, calcium storage, ROS, and activities of mitochondrial-regulated cell death signaling pathways. 

#### 3.1.8. Antioxidants

**Figure 3:**
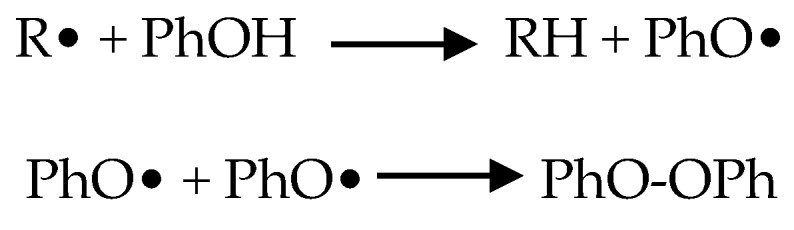
Polyphenolic neutralization of reactive oxygen species (ROS).

The studies selected for this review analyzed the abilities of antioxidants to counteract the toxic effects of arsenic on mitochondria in cardiovascular tissues and cells. It is worth noting that the majority of these antioxidants were phenolic compounds. Polyphenolic compounds belong to a heterogeneous group of chemicals that contain one or more aromatic rings and one or more hydroxyl groups. Polyphenols have non-enzymatic antioxidant activities that scavenge free radicals and oxidants such as O^−^, ONOO^−^ and H_2_O_2_. Oxidants are deactivated by polyphenolic antioxidant (POH) donation of a hydrogen atom, forming a phenoxy radical (PhO•). Phenoxy radical intermediates may stabilize either through intermolecular bonding between two polyphenols or via reaction with other radicals (Figure 3).

Polyphenols can be subcategorized as flavonoids, stilbenes, phenolic acids, and lignins (Table 2). Flavonoids are subcategorized as isoflavones, flavonols, flavones, flavanols, proanthocyanidins, and anthocyanins [93].

Antioxidants of both phenolic and non-phenolic classification with the ability to counteract pathophysiological effects of arsenic exposure in the cardiovasculature are listed and grouped by arsenic-induced effect in Table 3. 

Several studies investigated non-polyphenolic compounds. These included taurine, a sulfonic acid derivative of the amino acid cysteine; omega-3 fatty acid, which is found in fish oil and contains eicosapentaenoic acid (EPA) and docosahexaenoic acid (DHA); flax seed oil, an alpha-linoleic acid; and the organosulfur compound alpha-lipoic acid.

#### 3.1.9. Polyphenols

**Figure 4:**
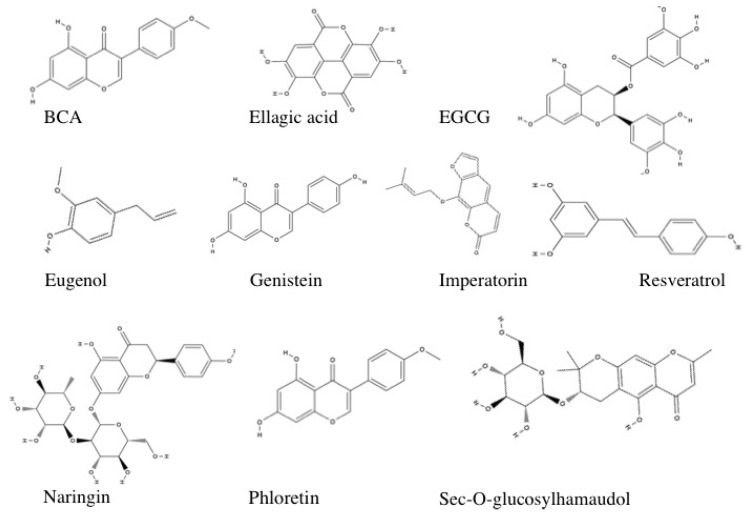
Polyphenolic antioxidant structures.

Representative stuctures of select polyphenolic compounds with demonstrated capaciy to ameliorate arsenic-induced cardiotoxicity are shown in Figure 4.

##### Biochanin A

Biochanin A (BCA) is found in red clover, cabbage, and alfalfa. BCA is a flavonoid polyphenol that scavenges free radicals and chelates and mobilizes toxins due to the presence of methoxy substitutions [55,94]. BCA is initially metabolized into genistein, but if excess BCA is available it is metabolized into both genistein and daidzein. Although daidzein is an isoflavone, it has relatively poor antioxidant properties and can induce oxidative stress by generating free radicals [55]. 

Sprague Dawley rats were orally treated with sodium arsenite at a concentration of 10 mg/kg/day or co-administered arsenic with BCA at 10, 20, or 40 mg/kg/day for 6 weeks [55]. BCA was unable to restore arsenic-induced elevations in MDA [55]. BCA at the 20 μM concentration significantly reduced SOD but was incapable of restoring GSH and catalase in heart tissue [55].

BCA significantly restored triglyceride levels, LDL and V-LDL cholesterol, atherogenic coefficient (AC)—a ratio of non-HDL cholesterol to HDL cholesterol, and cardiac risk ratio (CRR)—a ratio of non-HDL cholesterol to total cholesterol [55]. No pathological signs of arsenic toxicity were detected in heart tissues [55]. It is worth noting that although BCA was not effective at restoring all parameters, BCA alone had no negative effects on any variables in normal rats. The authors observed that BCA was only moderately effective at restoring arsenic-induced damages in cardiovascular tissues because of the pro-oxidative properties of the metabolite daidzein [55].

Although not the focus of the present study, BCA at 20 mg/kg and 40 mg/kg restored renal dysfunction, measured with urinary urea, creatinine, and BUN ratio. BCA at 20 mg/kg reduced MDA, GSH content, SOD activity, and catalase activity in kidney tissues and protected kidney architecture compared to arsenic-exposed tissue [55].

##### Boerhavia Diffusa

*Boerhavia diffusa* (BDE) is a potent antioxidant that contains biologically active polyphenols including retenoids and flavonoids as well as amino acids, lignins, saponins, b-sitosterols, and tetracosanoic, eicosanoic, stearic, and ursolic acids [73,95]. 

H9c2 cardiomyocytes were treated with 5 μM, 7.5 μM, and 10 μM arsenic in the presence or absence of 20 μg/mL BDE for 24-h. BDE reversed many of the effects of arsenic in H9c2 cardiomyocytes, particularly at the lower doses of arsenic tested. BDE (20 μg/mL) prevented alterations in morphology and cell viability caused by 5 μM and 7.5 μM arsenic [73]. BDE also increased the uptake of neutral red (NR) a supravital dye that viable cells incorporate in the lysosome- which had been reduced with 5 μM arsenic, and significantly rescued LDH release from cells treated with all arsenic concentrations [73]. BDE reduced ROS and oxidants (H_2_O_2_, OH-, ONOO-), and reduced morphological aberrations of apoptosis caused by exposure to 5 μM [73]. BDE restored transmembrane potential and reversed Ca^2+^ influx caused by exposure to 5 μM and 7.5 μM arsenic. Additionally BDE reversed alterations in lysosome, ER, and contractile protein morphology in cells exposed to 5 μM and 7.5 μM arsenic, and maintained ER integrity in cells exposed to 5 μM arsenic [73].

##### Ellagic Acid

Ellagic acid is a phenolic compound found in blackberries, raspberries, strawberries, cranberries, grapes, pomegranate, and walnuts [96]. Ellagic acid is considered a potent antioxidant and exhibits antibacterial, antiviral, anti-inflammatory, anti-fibrotic, anti-atherogenic, anti-mutagenic, and immunoregulatory properties [97]. The authors orally administered 30 mg/kg of ellagic acid to Wistar rats, followed onehour later by an intraperitoneal injection of 5 mg/kg arsenic for 10 days. Ellagic acid co-treatment increased QTc interval and decreased the cardiac biomarkers troponin-I and CK-MB [54]. Ellagic acid co-treatment significantly decreased GPx activity, and significantly decreased lipid peroxidation in the heart tissue of experimental animals [54]. Ellagic acid also prevented arsenic-mediated myofibrillar loss and myofibrillar coagulative necrosis [54].

##### EGCG

(−)-Epigallocatechin-3-gallate (EGCG) is a catechin and the most abundant flavonoid found in green tea [49]. EGCG has potent antioxidative activity because of the two triphenolic groups in its molecular structure [49] and has been used for the treatment of cancer, cardiovascular diseases, and autoimmune disease [98,99].

Sun et al. (2016) exposed Sprague-Dawley rats to sodium arsenite at a concentration of 50 mg/kg/day alone or in combination with EGCG at a concentration of 50 mg/kg/day for 30 days. Additionally, H9c2 cardiomyocytes were treated with 1 μM arsenic alone or with 1 μM EGCG for 24 h. 

EGCG fully reversed morphological changes in the myocardium including necrosis, intracellular edema, myofibrillar derangements, swollen and damaged mitochondria, and wavy degeneration of muscle fibers [49]. EGCG significantly reduced arsenic accumulation in the hearts of experimental rats and significantly inhibited arsenic-induced elevations in the activities of the cytoplasmic enzymes LDH, CK-MB, and AST in tissue [49]. 

EGCG co-treatment significantly reduced apoptosis, significantly increased the catalytic activities of SOD, catalase, and GPx, significantly decreased lipid peroxidation, and restored calcium balance [49]. In H9c2 cells, EGCG co-treatment significantly decreased LDH release, increased cell viability, and decreased apoptosis, caspase-3 activity, and the level of cleaved caspase-3. Additionally, EGCG significantly inhibited arsenic mediated mitochondrial ROS [49]. 

##### Eugenol

Eugenol is a phenolic monoterpene and member of the allylbenzene chemical class of compounds. Eugenol is extracted from clove and has antioxidant activity attributed to its methoxyphenolic structure [32]. 

Binu et al. (2017) orally exposed Wistar rats to 4 mg/kg arsenic with or without 5 mg/kg eugenol for 30 days. Electrocardiograph (ECG) readings performed on anesthetized rats demonstrated that arsenic prolonged QT interval and caused low heart rate, while co-treatment with eugenol significantly corrected these measures [32]. Eugenol co-treatment significantly decreased sodium and calcium tissue electrolytes, and increased potassium tissue electrolytes compared to arsenic exposed rats [32]. 

Eugenol significantly increased Ca^2+^-ATPase activity and decreased Na^+^/K^+^-ATPase activity compared to the arsenic treated group [32]. Co-treatment with eugenol also significantly decreased levels of the cardiac markers CK-MB and LDH, and increased GSH content, GST activity, and GPx activity [32]. 

Lipid peroxidation was significantly elevated in the arsenic treated group, and significantly restored from eugenol co-treatment. Eugenol co-treatment also significantly decreased arsenic deposition in the heart and restored structural abnormalities in the myocardium of arsenic treated rats to near normalcy [32]. Although the mechanism is not fully elucidated, the authors suggest that eugenol may trap free radicals to protect the myocardium from oxidative injury [32]. 

##### Genistein

Genistein is a natural biologically active flavonoid found in soy that has anti-cancer, anti-inflammatory and antioxidant properties [64]. Wistar rats were intravenously administered saline or genistein (10, 50, or 100 mg/kg/day) for 7 days, after which arsenic trioxide (0.8 mg/kg/day) was co-administered for another 7 days. Additionally, primary neonatal rat ventricular cells (NRVCs) were exposed to 10, 50 or 100 μM genistein for 1-h followed by incubation with 5 μM arsenic for various time points. 

Pre-treatment with genistein (10, 50, 100 μM) attenuated apoptosis in NVRCs exposed to 5 μM arsenic for 24-h [64]. Genistein also attenuated arsenic-induced up-regulation of JNK phosphorylation, phosphorylation of 38-MAPK (p38-MAPK), and cleaved caspase-3 in vivo and in vitro from exposure to 5 μM arsenic for 24-h [64]. In NVRCs, genistein dose-dependently attenuated the collapse of mitochondrial membrane potential induced with 12-h exposure to 5 μM arsenic, and DNA fragmentation induced with 24-h exposure to 5 μM arsenic [64]. In addition, pretreatment with genistein was effective against intracellular Ca^2+^ overload in NVRCs induced with 6-h exposure to 5 μM arsenic, and up-regulation of Cav1.2 (an alpha1 subunit of L-type calcium channel carrying IcaL) in vivo and in NVRCs following 6-h exposure to 5 μM arsenic. 

In vivo, genistein dose-dependently shortened arsenic-induced QT interval prolongation, and improved cardiac function impairment, including arsenic-induced reduced heart rate and reduced cardiac output in Wistar rats [64]. 

##### Grape Seed and Skin Extract

Grape seed and skin extract (GSSE) is a polyphenolic mixture containing flavonoids, stilbenes, proanthocyanidins, and other polyphenols [63]. The antioxidative effects of GSSG may be due to flavonoid components such as quercetin, which has independently been shown to reduce oxidative stress [100], resveratrol, a stilbene found in GSSE, or due to a synergism between polyphenols [63].

Wistar rats were exposed to 2.5 mg/kg of arsenic trioxide in the presence or absence of 4 g/kg GSSE for 21 days. Arsenic did not significantly alter body mass or heart mass of experimental animals, nor were total cholesterol or triglyceride levels affected. Alternately, arsenic exposure did provoke myocardial inflammation (measured with plasma CRP and LDH) and led to significantly elevated arsenic concentrations in plasma and heart tissue, while GSSE co-treatment significantly corrected the above disturbances to near control levels [63]. GSSE counteracted arsenic-induced increases in lipid peroxidation, carbonylation and non-protein sulfhydryl (NPSH) in the heart, all of which are promoted by ROS [63]. GSSG-co treatment also rescued catalase activity, GPx activity, and SOD activity to near control levels.

Interestingly, expression of the Cu/Zn SOD isoform was highly depressed in this study, while the Mn SOD isoform was unchanged. Thus the authors investigated the effect of arsenic on transition metals and determined that arsenic depleted copper in the heart, slightly increased zinc level in the heart, and had no effect on heart manganese [63]. In agreement with these findings, arsenic depressed the copper-dependent enzyme tyrosinase, slightly increased LDH (a zinc containing enzyme), and had no effect on the manganese-dependent glutamine synthase [63]. Heart copper deficiency is a recognized cause of myocardial fibrosis and heart failure. Copper deficiency has also been linked to cardiomyopathy, decreased cytochrome-c oxidase activity, hydrogen peroxide generation, and calcium dysregulation [101]. The authors suggest that the effect of arsenic on transition metal distribution (i.e., copper depletion and iron overloading), could be an initiation point of arsenic induced oxidative stress [63]. Importantly, GSSE significantly counteracted the effects of arsenic on transition metals and enzyme activities [63]. 

Compared to arsenic exposed rats, GSSE significantly decreased triglycerides, cholesterol, and lipase activity in the hearts of co-treated rats [63]. GSSE-co treatment also significantly decreased hydrogen peroxide, free iron, ionizable calcium, and calpain activity (a calcium dependent protease) [63]. GSSE was partially effective at correcting arsenic-induced effects on myocardial architecture such as decreased myocardial size, cytoplasmic vacuolization, myofibrillar loss, and mild swelling of fibers with mild interstitial edema [63]. 

##### Imperatorin and sec-*O*-glucosylhamaudol

*Radix Saposhnikoviae* is a traditional Chinese medicine from the root of *Saposhnikovia divaricata (Turcz.) Schischk* that contains the flavonoid components imperatorin and sec-*O*-glucosylhamaudol [61]. 

These two extracts were compared at a variety of concentrations for their ability to counteract the toxic effects of arsenic trioxide on cardiomyocytes. Hu et al. (2016) administered antioxidants 1-h prior to exposing cardiomyocytes to 2 μM/mL arsenic for 24-h. Both imperatorin (20, 30, 40 up to 90 μg/mL) and sec-*O*-glucosylhamaudol (60, 70, 80 μg/mL) protected against arsenic-induced cell death [61]. Fifty micrograms per milliliter of imperatorin or 50 μg/mL sec-*O*-glucosylhamaudol prevented LDH release. Imperatorin (100 μg/mL) and sec-*O*-glucosylhamaudol (50 μg/mL) prevented apoptosis, however, neither antioxidant decreased caspase-3 activity. Imperatorin (50 and 60 μg/mL) reduced ROS and oxidants (H_2_O_2_, HO-, and ONOO-), but sec-*O*-glucosylhamaudol failed to suppress ROS. Neither imperatorin nor sec-*O*-glucosylhamaudol significantly restored arsenic induced elevations in cellular calcium levels [61].

Compared to control cells, single exposure to arsenic, imperatorin, or sec-*O*-glucosylhamaudol increased mRNA expression of Nrf2, NQO1, and HO-1. Pretreatment of cells with imperatorin or sec-*O*-glucosylhamaudol, followed by arsenic exposure led to a further significant increase in mRNA levels of Nrf2 compared to arsenic treated cells. Additionally, pre-treatment with imperatorin (100 μg/mL) or sec-*O*-glucosylhamaudol (50 μg/mL) followed by arsenic exposure further significantly increased the expression levels of NQO1 mRNA [61]. In contrast, Imperatorin pre-treatment (50 μg/mL) significantly decreased HO-1, yet sec-*O*-glucosylhamaudol had no significant effect on HO-1 in combination with arsenic. 

In agreement with the effects of these antioxidants on mRNA expression, co-exposure to arsenic and imperatorin or sec-*O*-glucosylhamaudol significantly increased NQO1 protein levels. Again, imperatorin (75 μg/mL) co-treatment decreased HO-1 protein levels, whereas Sec-*O*-glucosylhamaudol co-treatment further significantly increased HO-1 protein levels. Although the authors do not offer an explanation for this apparent contradiction, they suggest that future studies should examine the significance of the downregulation of HO-1 mRNA and protein expression [61].

##### *Malus domestica* Apple Peel Extract

*Malus domestica* L. peel contains polyphenolic flavonoids such as anthocyanins, qurecetin glycosides and cyaniding glycoside [66]. *Malus domestica* L. peel has been reported to inhibit arsenic trioxide-induced LDL oxidation, reduce ROS, exhibit antihypertensive properties, and protect against damaged DNA and mitochondria [66]. Vineetha et al. (2014) investigated aqueous and methanolic extract of apple peel (40 μg/mL) against 5 μM arsenic in H9c2 cells exposed for 24-h (unless otherwise noted).

Both aqueous extract and methanolic extracts demonstrated high phenolic and flavonoid content [66]. Co-treating H9c2 cardiomyocytes with apple peel extracts effectively increased cell viability, decreased LDH release, and reduced morphological indications of apoptosis induced by arsenic trioxide. Apple peel extracts decreased mitochondrial superoxide, ROS, and oxidants (H_2_O_2_, OH-, and ONOO-). Apple peel extracts also reversed arsenic induced alterations in GPx activity to near control levels, reversed short term (6-h) increases in GSH content and longer term (≥12-h) decreases in GSH content.

Arsenic exposure significantly decreased SOD and catalase activity, while apple peel extracts significantly reversed these trends [66]. Apple peel extracts decreased XO activity, caspase-3 activity, and calcium levels, and increased TR activity to near similar levels as untreated cells [66].

##### Naringin

Naringin, a flavonoid antioxidant, is a polyphenolic compound found in citrus fruit that has anti-inflammatory, antioxidant, antihypertensive, and anti-cancer activities [70]. 

Adil et al. (2016) orally exposed Sprague-Dawley rats to 5 mL/kg arsenic for 28 days in the presence or absence of Naringin (20, 40, or 80 mg/kg). Naringin (40 and 80 mg/kg) increased heart weight, body weight, and heart rate, and decreased QRS, QT, QTc, and RR interval compared to arsenic-only treated rats [70]. Naringin (40 and 80 mg/kg) also significantly decreased systolic and diastolic blood pressures, and significantly decreased left ventricular end diastolic pressure (LVEDP) compared to arsenic-only treated rats [70]. 

Naringin (40 and 80 mg/kg) fully prevented edema, vacuolization, cytoplasmic eosinophilia, and inflammation, and partially reversed nuclear pyknosis, fibrosis, and collagen deposition in the myocardium of arsenic treated rats [70]. The higher concentrations of naringin significantly decreased serum levels of LDH, CK-MB, AST, ALT, ALP, total cholesterol, triglycerides, LDL and V-LDL, whereas serum HDL was significantly increased compared to arsenic-exposed rats [70]. Treatment with naringin at 40 and 80 mg/kg significantly increased SOD level, GSH level, and Na^+^/K^+^-ATPase activity, and significantly decreased MDA and NO content compared to the arsenic treated group [70].

Naringin co-treatment (40 and 80 mg/kg) significantly increased the activity at mitochondrial complexes I–IV and reduced the appearance of swollen and degenerated mitochondria with dilated perinuclear membrane and vacuolization [70]. Co-treatment with naringin (40 and 80 mg/kg) also restored arsenic-induced alterations in cardiac Nrf2 and HO-1, Smad-3 and TGF-β mRNA expression. Naringin (40 and 80 mg/kg) also decreased apoptosis and decreased ROS and oxidants (H_2_O_2_, OH^−^, and ONOO^−^) compared to arsenic treated rats [70].

##### Phloretin

Phloretin is a polyphenolic flavonoid found in apples and other sources. Vineetha et al. (2015) exposed H9c2 cells to 5 μM arsenic along with phloretin (2.5 and 5 μM). Both phloretin concentrations increased cell viability and decreased LDH release compared to arsenic exposed cells. Phloretin (2.5 and 5 μM) also restored arsenic-mediated increases in ROS and oxidants (mitochondrial superoxide and combined H_2_O_2_, OH^−^, and ONOO^−^) and arsenic- mediated reductions in GPx activity, GSH content, SOD activity, and TR activity. Co-treatment with phloretin at both concentrations decreased Nrf2 to levels near control cells [45]. 

Phloretin at both concentrations significantly restored arsenic-induced increases in XO and caspase-3 activity, and significantly restored arsenic-induced decrease in aconitase activity. Arsenic exposure significantly elevated calcium content and significantly reduced Ca^2+^-ATPase activity compared to control cells, whereas treatment with phloretin at both concentrations significantly restored these parameters [45]. 

Phloretin co-treatment significantly decreased mitochondrial superoxide levels compared to arsenic exposed cells, and phloretin restored activity in mitochondrial complexes I, III, and IV. In this study, arsenic did not result in significant changes in mitochondrial complex II. Phloretin restored arsenic-mediated decreases in ATP content, disruptions in transmembrane potential, and PTP pore opening, as well as arsenic-mediated reductions in oxygen consumption rate. Arsenic exposure also mediated mitochondrial swelling, which was restored with phloretin, and phloretin returned the percentage of necrotic and late apoptotic cells to near control levels [45].

##### Resveratrol

Resveratrol is a polyphenolic stilbene found in red wine. Zhang et al. (2013) studied the effects of resveratrol (8 mg/kg) against arsenic (3 mg/kg) in Wistar rats exposed every other day over an 8-day period. Arsenic exposure led to increased arsenic content in the hearts of Wistar rats, whereas resveratrol significantly reduced this accumulation. Compared to arsenic treated rats, rats co-treated with resveratrol demonstrated a significant decrease in markers associated with myocardial injury including LDH, CK, CK-MB and AST measured in plasma. In addition, resveratrol significantly reversed arsenic-mediated increases in the levels of ROS species and markers of oxidation (H_2_O_2_, OH-, ONOO-, 8-OHdG, and GSH/GSSG ratio). Resveratrol significantly reduced Ca^2+^ content compared to the arsenic treated group [33]. Resveratrol co-treatment partially restored arsenic-mediated myofibril loss, cardiomyocyte necrosis, and decreased cAMP levels in the heart [33]. Arsenic also mediated down regulation in Nrf2 and HO-1 mRNA gene expression in the heart, both of which were restored with resveratrol. In this study, there was no significant change in NO concentration in arsenic-treated or resveratrol-treated groups, possibly due to the short study duration [33].

Another study conducted by Zhao et al. (2008) tested resveratrol (3 mg/kg) against arsenic (1 mg/kg) exposure in BALB/c mice. Animals were exposed to arsenic intravenously on alternate days over a 6-day period. Mice co-treated with resveratrol received resveratrol injections 1-h prior to arsenic exposure. 

In vivo, resveratrol reduced QT elongation, decreased LDH activity in plasma, and increased catalase, GPx, and SOD activities compared to arsenic treated mice. Structural abnormalities following arsenic exposure (cytoplasmic vacuolization, myofibrillar loss, and cardiomyocyte necrosis) were partially prevented with resveratrol co-treatment [72]. Additionally, TUNEL-positive cells were detected with a greater frequency in the hearts of arsenic treated mice compared to control mice and this parameter was dramatically decreased by resveratrol treatment [72]. 

In vitro, H9c2 cardiomyocytes were pre-treated with 0.1, 1 or 10 μM Resveratrol, followed 1-h later by 10 μM arsenic for 24-h [72]. Resveratrol dose-dependently increased cell viability and dose-dependently reduced LDH release to the medium. Resveratrol dose-dependently reduced apoptosis and DNA damage, and pre-treatment with 10 μM resveratrol returned ROS and oxidant levels (H_2_O_2_, OH-, and ONOO-) to near control levels. Additionally, pre-treatment with 10 μM resveratrol significantly restored arsenic-induced increases in Ca^2+^ accumulation and Caspase-3 activity [72]. 

##### Resveratrol and Genistein

Fan et al. (2014) investigated the effects of the flavonoid genistein (50 μM) and the stilbene resveratrol (5 μM) on primary neonatal rat left ventricular myocytes (NRLVMs) exposed to arsenic (5 μM). Cardiomyocytes exposed to 5 μM arsenic for 12-h along with resveratrol or genistein demonstrated neutralized ROS and oxidants (H_2_O_2_, OH-, and ONOO-) generation and increased mitochondrial transmembrane potential. Resveratrol co-treatment reversed decreases in GSH while resveratrol and genistein co-treatment reversed the reduction in SOD activities induced with 24-h arsenic exposure. Additionally, genistein and resveratrol enhanced autophagy in 24-h arsenic treated cardiomyocytes. LC3, a marker of autophagy, was also measured due to the functional relationship between the mediators regulating oxidative stress and autophagy [102]. LC3 was elevated in resveratrol and genistein-arsenic groups compared to the arsenic treated group [65]. Therefore, an enhancement of autophagy in antioxidant-treated groups may represent a beneficial, compensatory response against oxidative stress by removing damaged organelles (mitochondria), whereas arsenic-treated groups demonstrated lower levels of autophagy and higher levels of apoptosis [65].

Moreover, resveratrol and genistein protected against apoptosis, DNA fragmentation, and decreased cell viability induced by a 24-h exposure to arsenic. When examined individually, a much lower dose of resveratrol (5 μM) was needed to achieve these effects in cardiomyocytes compared to genistein (50 μM). 

Although not the focus of the present review, Fan et al. (2014) also investigated the effects of arsenic and resveratrol/genistein in NB4 cancer cells. Arsenic causes cancer cell apoptosis by binding to protein kinase M2 (PKM2), located on the surface of PML/RARA. PKM2 promotes aerobic glycolysis (the “Warburg effect”) leading to tumorigenesis and cancer cell proliferation. Arsenic reduces PKM2 activity, thus inhibiting cancer cell growth [9]. It is worth noting that resveratrol/genistein increased the anti-cancer effects of arsenic in NB4 cancer cells by increasing ROS, enhancing mitochondrial transmembrane potential alteration, reducing GSH content and SOD activity, promoting apoptosis, and increasing autophagy beyond the levels achieved by arsenic alone [65].

##### Silybum Marianum

*Silybum marianum* (SB) is a polyphenolic flavonoid antioxidant of silymarin isolated from the seeds of milk thistle. SB has been used as a hepato-protective agent against arsenic induced liver toxicity [103] and has membrane stabilizing, anti-inflammatory, antioxidant, metal chelation, and cardioprotective qualities [104]. 

Wistar rats were exposed to 5 μM arsenic, 75 μM SB, or both for 4-weeks [103]. SB significantly decreased the activities of cardiac enzymes (CK-MB, LDH, AST, ALP), increased heart mitochondrial enzymes (ICDH, SDH, MDH, α-KDH, and NADH hydrogenase), decreased levels of plasma and cardiac lipids (cholesterol, triglycerides, and free fatty acids) and increased phospholipids compared to arsenic exposed rats [103]. Pre-treatment with SB significantly restored arsenic-induced increases in LDL cholesterol and V-LDL cholesterol, and restored arsenic-induced decrease in the level of HDL cholesterol in plasma. Markers of oxidative stress in the heart were significantly reduced by SB-co treatment, whereas GSH content, SOD activity, catalase activity, GPx activity, GST activity, GR activity, and G6PD activity were significantly increased compared to the arsenic treated group. SB partially reversed arsenic-induced changes in mitochondrial morphology and significantly reversed arsenic-induced decreases in the membrane bound ATPases Na^+^/K^+^-ATPase, Ca^2+^-ATPase, and mg^2+^-ATPase. SB co-treatment decreased the NOX2 and NOX4 protein levels. NOX family proteins mediate ROS, including H_2_O_2_ and superoxide, thus decreased NOX expression should decrease anti-oxidant responses. Indeed, SB co-treatment normalized arsenic-altered protein expression of Nrf2, HO-1, and Keap-1 in arsenic treated liver [103].

##### Sorbus Phnuashanesis (Hante) Hedl

Sorbus phnuashanesis (SPF) is a traditional Chinese herb with high flavonoid antioxidant activity. Yu et al. (2017) exposed BALB/c mice to 5, 10 or 20 mg/kg SPF by intraperitoneal injection followed one-hour later with 1 mg/kg arsenic by intravenous tail injection for 14-days. 

Arsenic caused structural abnormalities in heart tissue including cytoplasmic vacuolization, myofibrillar loss, and cardiomyocyte necrosis compared to control animals, whereas SPF pretreatment significantly alleviated these alterations [62]. ECG analysis demonstrated that SPF prevented arsenic-induced alterations in ST-T wave change and QT-interval prolongation. All SFP concentrations significantly reduced serum cardiac enzymes (CK, CK-MB, and LDH) compared to the arsenic treated group [62]. 

In vitro, H9c2 cells were pre-treated with 4 μM arsenic or co-treated with arsenic and 20 μg/mL SPF for 24-hs. SPF significantly maintained cell viability and reduced the release of LDH observed in cells exposed to arsenic for 24-h. Arsenic reduced the activities of SOD, catalase, and GPx in vivo and in vitro following 24-h exposure, whereas SPF significantly alleviated these oxidative stress responses [62]. In vitro, SPF significantly decreased arsenic mediated elevation in levels of H_2_O_2_, OH^−^ and ONOO^−^. Pre-treatment with SPF prevented arsenic-induced apoptosis in vitro and arsenic-mediated increases in the expression of caspase-3, caspase-8, and caspase-9 both in vivo and in vitro [62]. SPF also restored arsenic-induced reductions in Bcl-2/BAX protein ratios.

Akt plays a role in arsenic-induced apoptosis and can activate Nrf2. Arsenic significantly reduced the ratio of Akt phosphorylation to total Akt, but this was reversed by SPF co-treatment in H9c2 cells [62]. In agreement, Nrf2 expression was significantly reduced by arsenic, and significantly restored by SPF co-treatment. The Nrf2/antioxidant responsive element (ARE) signaling pathway can induce phase II detoxification enzymes including HO-1. Treatment by SPF was effective at significantly restoring arsenic induced downregulation of HO-1 [62].

##### Trichosanthes Dioca

*Trichosanthes dioca* (*T. dioica*) is a dioecious climber found in northern and northeastern India [68]. All parts of the plant have been used for medicinal purposes. The roots contain flavonoids, alkaloids, reducing sugars, saponins, and steroids [68], and the fruit contains flavonoids, glucosides, and alkaloids [67]. Bhattacharya et al. (2014) evaluated the hydroalcoholic extract of *T. dioica* root (TDA) against arsenic toxicity in vivo, and Bhattacharya et al. (2013) evaluated the aqueous extract of *T. dioica* fruit (AQTD) against arsenic toxicity in vivo.

Although these experiments are reported in separate journals, the experimental design was identical. In both sets of experiments, Wistar rats were orally administered water or the respective antioxidant (TDA at 5 or 10 mg/kg [68], or AQTD at 50 and 100 mg/kg [67]) every other day for 20 days. On day 21, sodium arsenite was orally administered at 10 mg/kg for 8 consecutive days. Arsenic significantly reduced heart and body weight whereas pretreatment with TDA or AQTD dose-dependently ameliorated these effects [68]. White blood cell (WBC) count significantly increased and δ-aminolevulinic acid dehydratase (ALAD), red blood cell (RBC) count, and hemoglobin significantly decreased in arsenic exposed rats, whereas TDA or AQTD significantly and dose-dependently restored these parameters toward control levels [68].

Pre-treatment with TDA or AQTD significantly and dose-dependently decreased CPK, LDH, and total serum cholesterol, and increased HDL cholesterol compared to arsenic treated rats [68]. TDA and AQTD pre-treatment reduced TBARS, increased GSH content and decreased GSSG content compared to arsenic treatment of myocardial tissues [68]. GST, GPx, GR, SOD, and catalase activities were significantly decreased in arsenic-treated rats and significantly modulated by TDA or AQTD in a dose related manner [68]. DNA fragmentation was prevented by TDA or AQTD treatment prior to arsenic exposure [68]. 

While both the fruit and roots of *T. dioca* were effective against arsenic-induced toxicity, it is worth noting that a significantly lower dose of TDA (5 or 10 mg/kg body weight) compared to AQTD (50 or 100 mg/kg body weight) was used to achieve similar results. 

#### 3.1.10. Other Antioxidants

**Figure 5:**
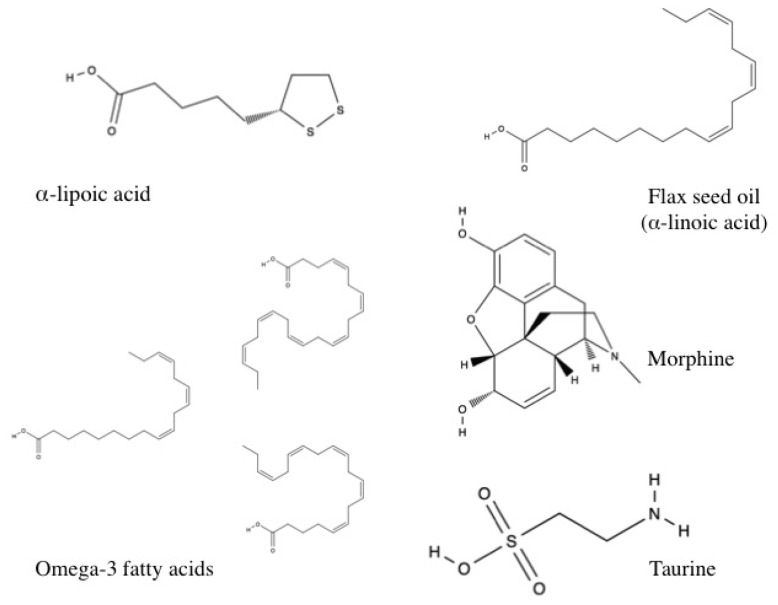
Structure of non-polyphenol antioxidants.

Select non-polyphenolic antioxidants bearing demonstrated cardioprotective effects against arsenic exposure are shown in Figure 5.

##### α-lipoic Acid

α-lipoic acid (LA) is an organosulfur compound derived from octanoic acid. LA contains two sulfur atoms connected by a disulfide bond and has antioxidant properties. Kumazaki et al. (2011) injected Wistar rats with 5 mg/kg/day arsenic for 8 weeks in the presence or absence of 35 mg/kg/day LA administered orally. Two of the 4 arsenic-exposed rats died suddenly at days 25 and 28 with no earlier symptoms, while all of the LA co-treated rats survived through the study [59]. Unexpectedly, LA did not prevent arsenic-induced decreases in body weight, decreased AST, or increased urinary excretion of 8-OHDG-an indicator of oxidative stress. This led Kumazaki et al. (2011) to hypothesize that LA prevented death without reducing oxidative stress, possibly through preventing sudden cardiac death [59]. To test this hypothesis, the authors exposed Wistar rats to 0.15, 1.5 and 5 mg/kg arsenic for 2-h followed by 70 mg/kg LA in order to assess the EKG profile [59]. Arsenic didn’t significantly alter QTc, but the highest arsenic dose caused transient ST-T wave change from 5 to 30 min post infusion, and prolonged the PQ interval [59]. LA prevented alterations in ST-T wave and PQ interval [59]. 

In a subsequent study, Kumazaki et al. (2013) further explored the cardioprotective effects and chelation potential of LA against QT interval prolongation in a guinea pig model [58]. Hartley guinea pigs were exposed to 1.5 mg/kg arsenic intravenously in the presence or absence of LA (0.35, 3.5, or 35 mg/kg). The experiment was repeated with LA post-treatment occurring one-h after arsenic treatment. Both sets of experiments exposed guinea pigs for a 2-h time frame and ECG readings were taken in live guinea pigs [58]. 

Continuous infusion with arsenic prolonged QTc interval as early as 60 min after dosing, and this effect was dose-dependently and significantly attenuated by LA co-treatment [58]. Post-treatment with LA also rapidly ameliorated QTc interval prolongation observed with arsenic exposure [58]. The authors suggest that arsenic may induce QT prolongation via the human ether-*a*-*go*-*go*-related gene (hERG) channel, thus, Kumazaki et al. (2013) looked at acute exposure on the slowly activating delayed rectifier K^+^ current (I_Ks_) in ventricular myocytes isolated for patch clamping. 

Cardiomyocyte exposure to 1 μM arsenic inhibited I_Ks_ currents and reduced amperage, both of which were rapidly restored to basal levels with 10 μM LA. After washout of LA, arsenic-induced I_Ks_ inhibition returned [58]. Decreased I_Ks_ current prolongs ventricular repolarization, and is one of the most important mechanisms involved in Torsades de Pointes [105]. 

LA and its reduced form dihydrolipoic acid (DHLA) bind metal ions, thus the chelating potential of LA was investigated with electrospray ionization time-of-flight (ESI-TOF) mass spectrometry analysis. When arbitrary concentrations of LA and arsenic trioxide were mixed, a peak at *m*/*z* 450.98 was detected indicating that one molecule of LA (MW 206.3) bound one molecule of As_2_O_3_ (MW 197.8) with solvent molecules (formic acid (MW 46.0) and protons (MW 1.0)). This peak was not present when the two molecules were analyzed separately [58]. 

##### Flax Seed Oil

Flax seed oil (FSO) is a rich plant source of polyunsaturated fatty acid alpha-linolenic acid (ALA), a precursor to eicosapentaenoic acid (EPA) and docosahexaenoic acid (DHA) which has anticancer, anti-inflammatory, and anti-atherogenic effects [106]. Wistar rats were exposed to 4 mg/kg/day arsenic in the presence or absence of 500 mg/kg FSO for 45-days [53]. FSO decreased arsenic deposition in the heart and restored structural changes in cardiac tissue compared to arsenic-treated rats [53]. Importantly, FSO significantly reduced CK-MB levels, LDH levels, and TBARS, and significantly increased GSH level and the activities of SOD, GPx, GST, and Catalase compared to arsenic treated rats [53]. 

##### Morphine

Morphine, an opioid, has antioxidant effects including ROS scavenger capacity, NADPH oxidase activity, and increased glutathione levels [107]. Pre-treatment with 1 μM morphine protected H9c2 cardiomyocytes against 24-h 0.5, 1, and 2 μM arsenic-induced decreases in cell viability, decreased activity at mitochondrial complex II, and increases in mitochondrial superoxide, and ROS and oxidants (H_2_O_2_, OH-, and ONOO-) [60]. Arsenic dose-dependently induced DNA damage, while morphine pre-treatment significantly prevented this deleterious effect [60]. Morphine significantly decreased caspase-3 activity induced with 24-h exposure to 1 and 2 μM arsenic [60]. Forty-eight h exposure to 2 μM arsenic significantly increased the expression of pro-apoptotic BAX and PUMA genes, and significantly decreased the expression levels of anti-apoptotic BCL-2, Survivin, CIAP1, CIAP2, XIAP, and BCL-XL, whereas morphine significantly reversed alterations in BAX, PUMA, BCL2, and Survivin [60]. Additionally, morphine pre-treatment significantly activated NF-κB compared to 0.5–2 μM arsenic treated cells [60]. 

Using morphine in clinical settings is controversial, particularly for chronic or non-cancer pain, based on concerns about addiction, safety, and efficacy [108]. Amini-Kohei et al. (2016) is the only report we are aware of using morphine to prevent arsenic induced cardiovascular toxicity, but it is worth noting that the antioxidant properties of morphine have been reported to protect against methyl-mercury intoxication in rat glioma cells [109] and reverse oxidative damage in neuroblastomas [110], astrocytes [111], and microglial cells [107]. Proposed mechanisms for the antioxidant properties of morphine include direct scavenger activity [107] recovery of GSH levels [110], and/or inhibition of NADPH oxidase activity [112]. Additionally, a recent study suggests that morphine induces cardioprotection by preventing oxidative stress through mitochondrial Src tyrosine kinase at mitochondrial complex 1 [113].

##### Omega-3 Fatty Acid

Omega-3 fatty acids, found in abundance in fish oil, are long chain polyunsaturated fatty acids with a common chain length of 18, 20, or 22 carbon atoms containing a C=C bond at the third carbon from the end, a carboxylic acid (COOH) at one end and a methyl (CH_3_) at the other end [57]. Omega-3s influence oxidative stress by inhibiting the production of inflammatory proteins, decreasing NF-κB activation, and reducing MAPKs [114]. Consuming omega-3 fatty acids, particularly eicosapentaenoic (EPA) and docosahexaenoic acid (DEA), decrease the risk of heart failure, and influence cardiac mitochondrial function by impacting membrane phospholipids [115]. Varghese et al. (2017) investigated the effect of omega-3 fatty acid (50 mg/kg) in vivo in Wistar rats exposed to 4 mg/kg arsenic for 45 days. 

Omega-3 co-treatment decreased arsenic deposition in heart tissues and decreased CK-MB [57]. Omega-3s also decreased lipid peroxidation, and increased GPx activity, GSH content, GST activity, SOD activity, and catalase activity compared to arsenic-treated groups [57]. Omega-3 co-treatment reduced the observed frequency of micronuclei and the presence of abnormal cardiac structures such as swelling near the epicardium, endocardium, and interstitial edema [57]. DHA (100 μM) protected against 24-h, 10 μM arsenic-induced LDH release, lipid peroxidation, intracellular Ca^2+^ accumulation, and alterations in MMP in cardiomyocytes [57].

##### Selenium

Selenium is an essential trace element present in legumes at concentrations that vary based on soil selenium levels. Selenium and arsenic interact metabolically, resulting in biliary excretion as seleno-bis-*S* (glutathionyl)-arsenium ion ([GS)2AsSe]-) [71]. Krohn et al. (2016) exposed APO E-/- mice to selenium deficient (0.009 mg/kg), selenium adequate (0.16 mg/kg), or selenium fortified (0.3 mg/kg) diets for 2 weeks, followed by 3 weeks of concurrent exposure to 200 ppb sodium arsenite in drinking water [71]. Arsenic exposure significantly increased atherosclerotic plaque formation in the aortic arch of selenium deficient and selenium adequate mice, whereas arsenic exposed mice in the selenium-fortified group demonstrated reduced plaque formation that resembled selenium-fortified controls [71]. Arsenic exposure in conjunction with selenium deficiency led to increased macrophage content in plaques, indicating decreased plaque stability [71]. Arsenic also increased oxidative stress, measured as decreased concentration of hepatic GSSG, whereas selenium-containing diets significantly restored hepatic GSSG [71]. Arsenic exposure in conjunction with a selenium-deficient diet decreased HDL to LDL ratio compared to the two other diets. 

Krohn et al. (2016) report no significant effects of arsenic on total serum cholesterol, LDL cholesterol, triglyceride level, or TBARS. However, the authors report a significant effect of diet on numerous endpoints regardless of arsenic exposure. For example, selenium deficiency increased lesion area in control and arsenic exposed mice. Furthermore, total cholesterol, LDL, and triglycerides were increased in selenium deficient groups regardless of arsenic exposure. This study highlights the negative effects of selenium deficiency alone on cardiovascular health, which resulted in increased plaque formation, as well as the exasperation of plaque formation in the arsenic exposed and selenium deficient condition [71]. 

##### Taurine

Taurine is a sulfonic acid derivative of the amino acid cysteine, which is neither a classic scavenger of ROS nor a regulator of antioxidant defenses [69]. Rather, taurine is believed to serve as a regulator of mitochondrial protein synthesis, which enhances the electron transport chain and protects mitochondria against excessive superoxide generation [104,116]. Taurine is involved in ion channels, transporters, and enzymes, modulates intracellular calcium [117], and has antioxidant properties [118].

Zhao et al. (2008) exposed Wistar rats to 2 mg/kg arsenic for 5 weeks, or 50 mg/kg taurine for 2 weeks followed by 2 mg/kg arsenic for 5 weeks. The in vitro component of this study involved exposing neonatal cardiomyocytes to 25 mM taurine, 5 μM arsenic, or 25 mM taurine followed by 5 μM arsenic (after 1-h) for 24-h. Overall, taurine increased SOD, catalase, GST, GR, and GPx activities, and increased GSH level and GSH/GSSG ratio compared to the arsenic treated group [69]. Taurine significantly decreased total cholesterol, MDA, and LDH compared to the arsenic treated group, and increased the level of HDL cholesterol to near control levels [69]. Taurine also improved cell viability and decreased apoptosis compared to arsenic-treated rats [69].

Taurine decreased NF-κB pathway phosphorylation [69]. Confirming the involvement of the NF-κB pathway in apoptosis, pre-incubation with the IKK inhibitor PS-1145 prevented arsenic-induced phosphorylation, caspase-3 activation, PARP cleavage, and cardiomyocyte apoptosis [69]. Oxidative stress can activate MAP Kinases as well as NF-κB, as MAPKs are mediators of cell death due to apoptosis [69]. The protein content of the MAPKs p38 and p-JNK, and to a lesser extent PERK, were elevated in heart tissue and cardiomyocytes following arsenic exposure, and taurine significantly decreased these parameters [69]. Pre-treatment with p38 and p-JNK inhibitors prevented arsenic induced NF-κB activation, suggesting that MAPK activity contributes to NF-κB activation [69]. 

Arsenic upregulated pro-apoptotic proteins BAX and Bad, and downregulated anti-apoptotic proteins Bcl_2_ and Bcl-xL, whereas taurine mitigated these effects [69]. Taurine increased mitochondrial membrane potential, reduced cytosolic cytochrome c content, and decreased intracellular Ca^2+^ compared to arsenic exposed groups [69]. 

## 4. Discussion

Arsenic impairs all aspects of mitochondrial function and mitigates apoptosis by elevating ROS. The antioxidants presented in this review largely prevented arsenic-induced pathology in vivo and in vitro. Compared to arsenic treatment alone, co-treatment with phytonutrient antioxidants restored cardiac function, reduced ROS levels, restored antioxidant activities, reduced apoptosis, reduced calcium overload, restored ATP content, and restored the activity of mitochondrial complexes. While particularly toxic to mitochondria, arsenic exposure also led to structural abnormalities in the endoplasmic reticulum, lysosomes, and contractile proteins [73]. It is worth noting that antioxidants were reported to be partially effective at restoring arsenic-induced alterations in the morphology of mitochondria and other organelles [70]. 

### 4.1. Identifying Inconsistencies

Several inconsistencies are worth mentioning. In one study arsenic caused a short-term increase in GSH [66], yet arsenic decreased GSH in all other instances [70]. Since GSH was also reduced at 12 and 24-h [66], the short term increase was likely a compensatory, beneficial mechanism in response to a specific arsenic dose and time.

Similarly, one study reported increased GPx activity after exposing Wistar rats to 5 mg/kg arsenic for 10 days [54]. This contrasts with the majority of studies in which arsenic decreased GPx activity [68]. It is possible that increased GPx activity reported by Hemmati et al. (2017) resulted from the relatively short exposure time (10 days) compared a much longer average exposure time of 25.4 days in other in vivo studies that investigated this endpoint. 

Additionally, one in vitro study reported arsenic-induced increase in Nrf2 level in conjunction with increased HO-1 and increased NQO1 [61]. In contrast, the majority of studies used an in vivo model and reported decreased Nrf2 level and decreased level of HO-1 [70]. It is possible that a difference in the in vitro cell culture environment caused this variability in the Nrf2 pathway [61]. Interestingly, past research has demonstrated that arsenic-induced effects often fail to follow a typical dose-response pattern [119,120]. This supports the hypothesis that the increase in antioxidant-related pathways is not an inconsistency due to technical variations but a true bimodal response that is dependent on dose and time of exposure to arsenic that can lead to increased upregulation of compensatory pathways. In agreement, past studies of various cell types have reported arsenic-induced upregulation of the Nrf2 pathway [76,77,78,79,80]. 

In addition to testing antioxidants against arsenic, all of the studies included in this review reported on the impact of antioxidants in the absence of arsenic (untreated conditions) to examine any potential beneficial or detrimental effects on cells/animals exposed to antioxidants alone. As expected, no significant positive or negative effects of antioxidants were reported, with the notable exception of imperatorin and sec-*O*-glucosylhamaudol, which upregulated the Nrf2 pathway. This is likely a compensatory response to stimulate additional antioxidant responses, as the Nrf2 pathway contains electrophile DNA elements that can lead to upregulation in the presence of oxidative stress [121]. 

### 4.2. Study Comparisons

Based on differences in cell type, experimental animal model, arsenic dose, duration, and experimental design, a direct comparison of antioxidants across studies is not possible in the present review. Only one study compared the effects of different antioxidants (resveratrol vs. genistein) in vitro and determined that resveratrol requires a lower dose for the same efficacy against 5 μM arsenic [65]. We can make a similar comparison between TDA and AQTD, since the same experimental setup was used by Bhattacharya et al. (2013) and Bhattacharya et al. (2014), revealing that a 10× lower concentration of TDA was effective against 10 mg/kg arsenic. Additionally, studies conducted by Adil et al. (2016) and Muthumani et al. (2014) used the same arsenic-dosing paradigm and numerous overlapping endpoints. Thus, we can conclude that 40 mg/kg naringin was as effective as 75 μM silybum marianum at reversing the effects of 5 mg/kg sodium arsenite for the endpoints of total cholesterol, LDL, LDH, AST, ALT, CK-MB, Nrf2, HO-1, SOD activity, GSH activity, NA^+^/K^+^ ATPase, and altered mitochondrial morphology [70]. In order to improve our understanding of the capacity of antioxidants to prevent arsenic induced dysfunction, an in vivo investigation should be conducted on the pharmacokinetics of arsenic and antioxidants in the heart in relation to cardiotoxicity.

In vitro, there was some overlap in experimental cell type, arsenic concentration, and endpoint, and we can categorize in vitro studies conducted on cardiomyocytes by low, moderate, and high arsenic exposure, defined as <2 μM, 2 to 5 μM and ≥5–10 μM. We suggest that a more extensive comparative study be conducted on H9c2 cardiomyocytes testing polyphenols at their optimized concentrations against an intermediate dose of 5 μM arsenic.

### 4.3. Limitations

Despite the overall strength of the studies included in this review, we acknowledge a level of variability in the strength of study design and data reporting of included studies (Table S1). All included studies were peer reviewed, had clearly stated experimental objectives, and all in vitro studies identified the source and/or isolation method of cells. Among in vivo studies, 92% fully disclosed the characteristics of the experimental model including animal species, source, gender, and physical characteristics. In addition, 68.4% of studies analyzed in this review specified that animals were randomly assigned to experimental groups, and all studies used commonly accepted experimental methods.

However, no studies specifically conducted a power analysis to justify the size of experimental groups or the number of technical replicates in experiments. Additionally, no studies reported effect sizes. In the absence of effect sizes, the relevance of antioxidant effects cannot be comparatively assessed. 

With regard to data assessment and reporting, 88% fully reported the statistical methods used, and 91% of these studies used appropriate methods. 

The average number of citations per article was 12.1 ± (20.5), and the average H index (a measure of productivity and citation impact) for journals in which articles were published was 86.8 ± (50.8).

While several aspects of these studies are lacking, particularly in the area of complete data reporting, our results of the studies analyzed provide compelling evidence that antioxidants protect against arsenic-induced mitochondrial dysfunction, and make a strong case for performing additional studies that directly compare the efficacy of different antioxidants.

A potential limitation for the applicability of this research in a real world context is low bioavailability of polyphenol antioxidants. Polyphenols are defined and categorized based on the nature of their chemical skeletons. Major classes are phenolic acids, flavonoids, stilbenes, and lignins. Flavonoids are the most abundant polyphenol in our diets, and classes of flavonoids are based on the degree of oxidation of the oxygen heterocycle [93,122]. Flavonoid classes are isoflavones, flavonols, flavones, flavanols, proanthocyanidins, and anthocyanins [93].

Assessing polyphenol bioavailability is not straightforward. Measuring polyphenol antioxidant quantities is a useful way to compare food items, yet polyphenol content cannot necessarily be extrapolated to bioavailability or health claims [123]. For example, consuming flavonoid-rich food will only slightly increase the flavonoid content of plasma due to the process of polyphenolic metabolism, in which flavonoids are broken down into smaller phenolic acids, absorbed and metabolized to form glucuronides and sulfide conjugates [123]. Conjugated forms of polyphenols appear in circulation and can have altered bioactivity from the parent compound [123].

Individual variability in levels of the enzymes involved in polyphenol metabolism also impact overall bioavailability of polyphenols [123]. Some polyphenols are absorbed in the stomach or small intestine, while others are absorbed in the colon following metabolism by gut microflora. Variations in gut microbe populations further alter polyphenol bioavailability at the individual level [123].

Despite challenges predicting polyphenol bioavailability, studies have reported on concentrations of polyphenols circulating in plasma, or the amount of polyphenol measured in urine, even though these observations do not necessarily correlate with the amount of a compound that reaches a target site to exert a desired effect [123]. Although an indirect method to assess bioavailability, a more accurate measure of polyphenol bioavailability is to assay for markers of peroxidative damage in vivo following polyphenol consumption. However, a limited number of studies on polyphenol bioavailability have been conducted using this method in humans. The available data suggests that among flavonoids, isoflavones have the highest bioavailability, with absorption between 33 and 100%, followed by flavonols (12–41%), and flavanones (11–16%) [122]. Tea catechins and anthocyanins are among the polyphenols with the lowest absorbability, while catechins have intermediate absorption characteristics (lower than isoflavones and higher than flavonols [123]) 

An additional potential limitation on applying these findings in arsenic-affected regions relates to the ability to extrapolate in vitro and animal model experimental results to human populations. The dose ranges of both arsenic and ameliorative antioxidant treatments employed in the reviewed studies vary significantly. Although the doses of arsenicals used in these studies may be considered high, they are applicable in the context of human exposures. Humans have been acutely and chronically exposed to similar concentrations of arsenicals environmentally [124] and in the clinical treatment of acute promyelocytic leukemia [125]. Furthermore many epidemiological studies have demonstrated the pathological effects of long-term accumulation of arsenicals in human bodies at high doses [3,126].

We also acknowledge a lack of consensus in the research on the benefits of antioxidant supplementation in humans. Observational studies suggest that consuming greater amounts of antioxidant-rich foods decreases the risks of cardiovascular diseases, stroke, and cancer, but it is not entirely clear if these effects are due to antioxidants themselves, due to other substances in fruit and vegetables, or a result of other behaviors (such as exercise) among individuals who consume a higher volume of antioxidant-rich foods.

While laboratory studies consistently demonstrate that antioxidants stabilize free radicals, many long-term clinical trials of antioxidant supplementation have failed to conclusively demonstrate a benefit of antioxidant supplementation. It is worth noting, however, that several recent double blind, placebo-controlled studies support the role of antioxidant amelioration of oxidative stress in humans. For example, 12-week supplementation with 100 mg resveratrol significantly ameliorated arterial stiffness and decreased serum diacron-reactive oxygen metabolites (d-ROMS)-a measure of oxidative stress- in type 2 diabetics [107], supplementation with 500 mg/day resveratrol significantly decreased oxidative stress (measured as serum MDA) in subjects with ulcerative colitis [108], and supplementation with α-lipoic acid in type-2 diabetics significantly increased SOD and GPx, and decreased MDA in subjects compared to baseline measures and compared to the placebo-control group [109]. Thus, recent clinical trials provide evidence that antioxidant’s ability to ameliorate oxidative stress translates to human subjects.

Additionally, several clinical trials have reported mild benefits of selenium pill supplementation on measures of arsenic toxicity [127,128,129], and Krohn et al. (2016) are currently planning a clinical trial supplementing the diet of arsenic-exposed individuals in Bangladesh with selenium fortified lentils [130].

### 4.4. Future Directions

Given the limitation of low polyphenol bioavailability, research has been conducted on combining polyphenols to improve bioavailability. Evidence suggests that combined polyphenols can have a synergistic effect, particularly with regard to anti-cancer properties. For example, combining EGCG and curcumin allowed for an 8-fold dose reduction in EGCG in the mixture for the same therapeutic effect [120]. Additionally, Fan et al. (2014) reported that the combination of resveratrol and genistein was more effective against NB4 cancer cells than either polyphenol alone [65]. Therefore, based on these aforementioned observations in cancer, it is conceivable that combination therapies involving more than one polyphenol can exert synergistic effects in reversing cardiovascular pathology following arsenic exposure.

Another promising area of research is on improved polyphenol extraction and preservation techniques. Polyphenols are found in many natural sources complexed with sugars or proteins, or in polymerized derivatives [120]. To achieve effective isolation, proper selection of a solvent for extraction is necessary to maintain molecular integrity, and recovery depends on time and temperature (high temperature, long extraction times, and alkaline environment cause degradation) [120]. 

More recently, encapsulation and nanoformulation have been proposed as potential solutions, as these techniques can improve both stability and bioavailability of polyphenols [120]. Nanoformulations include nanosuspensions, solid lipid nanoparticles, liposomes, gold nanoparticles, micelles, and polymeric nanoparticles. Nanoencapsulaton can increase intracellular concentration and allow slow and sustained polyphenol release [131].

Nanoformulations have been developed for several polyphenols for use against cancer cells in pre-clinical trials. Ellagic acid encapsulated nanoparticles were developed for use in oral cancer with improved results [132]. In addition, EGCG nanoformulations have been used against human prostate cancer cells, human melanoma cells, mouse models of melanoma, breast cancer cell lines, and breast cancer cells isolated from patients [133]. Silibinin nanoparticles have been used against human hepatocellular cells and carcinoma cell lines [134], and resveratrol nanoformulations have been used against human prostate cancer cell lines [135], murine melanoma cells [136], rat glioma cells [137], human head and neck cancer cells [138], and human ovarian cells [139]. Importantly, resveratrol has also been developed into a mitochondrial targeting drug to improve anti-cancer ability [140]. Sassi et al. (2014) linked resveratrol derivatives with an o-linked mitochondria targeting 4-triphenlyphoshponlumbutyl group to selectively kill fast growing cells [140]. Additionally, a mitochondrial targeted prototype nanoformulation of genistein was developed by Pham et al. (2013) and is reported to increase cytotoxicity through the intrinsic apoptotic pathway against hepatic and colon carcinoma [141]. 

Although not focused specifically on arsenic-mediated cardiovascular dysfunction, several recent clinical trials have assessed the benefit of polyphenols on general cardiovascular effects. For example, Richter et al. (2017) reported that isoflavone-containing soya protein supplementation significantly reduced brachial diastolic blood pressure compared to a lower dose of soya protein (but not compared to control) [142]. Even more compelling, Tenore et al. (2017) demonstrated that supplementation with microencapsulated annurca apple polyphenol extracts, trade-marked as ‘AppleMetS’ significantly decreased LDL cholesterol, significantly increased HDL cholesterol and significantly decreased total cholesterol compared to control subjects in a randomized parallel group placebo-controlled 2 week study [131]. 

The present review identifies 21 compounds with antioxidant properties that are effective against arsenic-mediated mito-toxic effects in cardiovascular tissue. We believe that the current limitations in bioavailability of polyphenol antioxidants are surmountable. Also of interest, mitoquinone (MitoQ) is a mitochondrially targeted molecule designed as an antioxidant to block mitochondrial oxidative damage [143]. MitoQ selectively accumulates in mitochondria and is currently being pursued as a therapy for degenerative conditions such as Parkinson disease [144]. While MitoQ has not been tested against arsenic-induced cardiotoxiciy, it has demonstrated cardioprotective properties against Doxorubicin (DOX), which is used for treating various cancers despite severe cardiovascular side effects including congestive heart failure, arrhythmias, and cardiomyopathy [145]. Importantly, mitochondria-targeting nanoformulations represent a compelling avenue of research for preventing arsenic-induced cardiovascular toxicity in a clinical context and among individuals chronically exposed to arsenic through ground water or APL therapy.

As a preliminary step, we suggest a pre-clinical approach of comparing antioxidant abilities of several combinations of polyphenol antioxidants to prevent arsenic-induced mito-toxic effects in H9c3 cardiomyocytes, while simultaneously increasing toxicity to NB4 cells. We suggest a focus on the mitochondrial endpoints of the Nrf2 pathway and associated antioxidants, caspase activation, apoptosis and associated Bcl-2/BAX expression, GSH activity, mitochondrial and overall ROS, calcium overload, Na^+^/K-ATPase activity, activity at mitochondrial complexes, OCR, and ATP generation. We believe the aforementioned proposed studies will give a comprehensive view of antioxidant capacity to prevent mitochondrial dysfunction. Following antioxidant selection, we recommend developing a mitochondrial-targeted nanoformulation for use in H9c3 and NB4 cell lines and Wistar rat models. As mitochondrial targeting nanoformulations of resveratrol and genistein have already been developed, adapting these formulations for use against arsenic-induced cardiotoxicity may be the most time- and resource-effective approach to developing real world solutions. 

## 5. Conclusions

Antioxidants have the potential to improve the cardiovascular health of millions of people chronically exposed to elevated arsenic concentrations through contaminated water supplies or used as lifesaving cancer treatments. While consuming foods containing antioxidants is a healthful and useful practice for the prevention of cardiovascular disease, additional research is needed on proper compounding, encapsulation and nanoformulations that prevent antioxidant degradation and improve polyphenol pharmacokinetics in order to counteract arsenic-induced cardiovascular toxicity in a real-world context. 

## Figures and Tables

**Table 1:** In vivo and in vitro study design.

**In Vivo Studies Stratified by Arsenic Exposure Duration**					
**Duration**	**Arsenic Dose**	**Type**	**Method**	**Model**	**Citation**
2 h	0.15 μM, 1.5 μM, 5 μM	As_2_O_3_	IV ^1^	Wistar rat	[59]
	1.5 μM	As_2_O_3_	IV	Guinea pig	[58]
6–10 days	0.8 mg/kg	As_2_O_3_	IV	Wistar rat	[64]
	1 mg/kg	As_2_O_3_	IV	Balb/c mouse	[72]
	3 mg/kg	As_2_O_3_	IV	Wistar rat	[33]
	10 mg/kg	NaAsO_2_	Oral	Wistar rat	[68]
	10 mg/kg	NaAsO_2_	Oral	Wistar rat	[67]
10–29 days	1 mg/kg	As_2_O_3_	IV	Balb/c mouse	[62]
	2.5 mg/kg	As_2_O_3_	IP ^2^	Wistar rat	[63]
	5 mg/kg	As_2_O_3_	IP	Wistar rat	[54]
	5 mg/kg	As_2_O_3_	Oral	SD rat ^3^	[70]
	5 mg/kg	NaAsO_2_	Oral	Wistar rat	[56]
	200 ppb	NaAsO_2_	Oral	APO E-/-	[71]
30–56 days	2 mg/kg	NaAsO_2_	Oral	SD rat	[69]
	4 mg/kg	As_2_O_3_	Oral intubation	Wistar rat	[57]
	4 mg/kg	As_2_O_3_	Oral intubation	Wistar rat	[57]
	4 mg/kg	As_2_O_3_	Oral	Wistar rat	[32]
	5 mg/kg	As_2_O_3_	IV	SD rat	[59]
	10 mg/kg	NaAsO_2_	Oral	SD rat	[55]
	50 mg/kg	NaAsO_2_	Oral	SD rat	[49]
**In Vitro Studies**					
	1 μM	NaAsO_2_		H9c2 ^4^	[49]
	1 μM, 2 μM	As_2_O_3_		H9c2	[60]
	2 μM/mL	As_2_O_3_		H9c2	[61]
	4 μM	As_2_O_3_		H9c2	[62]
	5 μM	As_2_O_3_		NRLVM ^5^	[65]
	5 μM	As_2_O_3_		H9c2	[66]
	5 μM	As_2_O_3_		H9c2	[45]
	5 μM	NaAsO_2_		Primary myocytes	[69]
	5 μM, 7.5 μM, 10 μM	As_2_O_3_		H9c2	[73]
	5 μM, 6 μM, 12 μM	As_2_O_3_		NRLVM	[64]
	10 μM	As_2_O_3_		H9c2	[57]
	10 μM	NaAsO_2_		H9c2	[72]

^1^ Intravenous; ^2^ intraperitoneal; ^3^ Sprague Dawley; ^4^ H9c2-rat heart cardiomyocyte cells; ^5^ neonatal rat left ventricular myocytes.

**Table 2:** Antioxidant classification and source.

**Polyphenolic Antioxidants**	**Classification**	**Source**
Biochanin A [55]	Flavonoid	Cabbage, alfalfa
*Boerhavia diffusa* [73]	Flavonoid	*B. diffusa*
Ellagic acid [54]	Phenol	Berries, walnuts
EGCG [49]	Catechin	Green tea
Eugenol [32]	Phenol	Clove
Genistein [65]	Flavonoid	Soy
Grape seed and skin extract [63]	Flavonoid, stilbene	Grapes
Imperatorin [61]	Flavonoid	*Radix Saposhininkovaie*
Sec-*O*-glucosylhamandol [61]	Flavonoid	*Radix Saposhininkovaie*
*Malus domestica* apple peel [66]	Flavonoid	Apples
Naringin [70]	Flavonoid	Citrus fruit
Phloretin [45]	Flavonoid	Apples
Resveratrol [33]	Stilbene	Red wine
*Silybum marianum* [56]	Flavonoid	Milk thistle
*Sorbus phnuashanesis (Hante) Hedl* [62]	Flavonoid	Chinese herb
*Trichosanthes dioca* [68]	Flavonoid	*T. dioica*
**Other Antioxidants**	**Type**	**Source**
α-lipoic acid [59]	Organosulfur compound	Spinach, broccoli
Flax seed oil [53]	α-linoleic acid	Flax seeds
Morphine [60]	Opioid	Poppy seeds
Omega-3 fatty acid [57]	Polyunsaturated fatty acid	Fish oil
Selenium [71]	Essential trace element	Lentils
Taurine [69]	Sulfonic acid	Amino acid cysteine

**Table 3:** Arsenic induced effects and antioxidant restoration.

Arsenic Induced Effect	Restored with Antioxidant	Citation
**Heart**		
Arsenic deposition in heart	Eugenol	[32]
	Grape seed and skin extract	[63]
	EGCG	[49]
	Omega-3	[57]
	Flax seed oil	[53]
	Resveratrol	[33]
QT interval prolongation	Naringin	[70]
	Eugenol	[32]
	Genistein	[64]
	α-lipoic acid	[58]
	Sorbus phnuashanesis	[62]
	Resveratrol	[72]
Increased QTc interval	Naringin	[70]
	Ellagic acid	[54]
	α-lipoic acid	[58]
ST-T wave change	Sorbus phnuashanesis	[62]
Increased RR interval	Naringin	[70]
Increased QRS interval	Naringin	[70]
Inhibited I_KS_ currents	α-lipoic acid	[58]
Reduced amperage of I_K_	α-lipoic acid	[58]
Decreased heart rate	Naringin	[70]
	Eugenol	[32]
	Genistein	[64]
Decreased cardiac output	Genistein	[64]
Decreased CAMP	Resveratrol	[33]
Structural changes in cardiac tissue	Naringin	[70]
	Eugenol	[32]
	Ellagic acid	[54]
	Grape seed and skin extract ^1^	[63]
	EGCG	[49]
	Omega-3	[57]
	Flax seed oil	[53]
	Sorbus phnuashanesis	[62]
	Resveratrol ^1^	[33]
	Resveratrol ^1^	[72]
Atherosclerotic plaque formation	Selenium	[71]
Increased ALP activity	Naringin	[70]
	*Silybum marianum*	[56]
**Blood Plasma**		
Elevated triglycerides	Biochanin A	[55]
	Grape seed and skin extract	[63]
Increased total cholesterol	Naringin	[70]
	*T. dioica* root	[68]
	*T. dioica* fruit	[67]
	Taurine	[69]
	*Silybum marianum*	[56]
	Grape seed and skin extract	[63]
Increased LDL cholesterol	Naringin	[70]
	Biochanin A	[55]
	*Silybum marianum*	[56]
Decreased HDL cholesterol	*T. dioica* root	[68]
	*T. dioica* fruit	[67]
Decreased phospholipids	*Silybum marianum*	[56]
Increased atherogenic Coefficient (AC)	Biochanin A	[55]
Increased cardiac risk ratio	Biochanin A	[55]
Increased free fatty acids	*Silybum marianum*	[56]
Increased lipase activity	GSSE	[63]
Increased CPK	*T. dioica* root	[68]
	*T. dioica* fruit	[67]
Increased CK-MB	Naringin	[70]
	Eugenol	[32]
	Ellagic Acid	[54]
	*Silybum marianum*	[56]
	EGCG	[49]
	Omega 3	[57]
	Flax seed oil	[53]
	Sorbus phnuashanesis	[62]
	Resveratrol	[33]
Increased serum troponin	Ellagic acid	[54]
Elevated LDH	Naringin	[70]
	*T. dioica* root	[68]
	*T. dioica* fruit	[67]
	Eugenol	[32]
	Biochanin A	[55]
	*Silybum marianum*	[56]
	EGCG	[49]
	Flax seed oil	[53]
	Sorbus phnuashanesis	[62]
	Resveratrol	[33]
	Resveratrol	[72]
Increased AST activity	Naringin	[70]
	α-lipoic acid	[59]
	*Silybum marianum*	[56]
	EGCG	[49]
	Resveratrol	[33]
Increased ALT activity	Naringin	[70]
	*Silybum marianum*	[56]
Increased ALP activity	*Silybum marianum*	[56]
Increased CRP	Grape seed and skin extract	[63]
Increased CK	Sorbus phnuashanesis	[62]
	Resveratrol	[33]
**Antioxidants**		
Downregulated NRf2	Naringin	[70]
	*Silybum marianum* (liver)	[56]
	Sorbus phnuashanesis	[62]
	Resveratrol	[33]
Increased Nrf2 level	sec-*O*-glucosylhamaudol ^2^	[61]
	Imperatorin ^2^	[61]
Upregulation of Keap-1	*Silybum marianum* (liver)	[56]
Decreased SOD activity	Naringin	[70]
	*T. dioica* root	[68]
	*T. dioica* fruit	[67]
	Resveratrol	[65]
	Genistein	[65]
	Taurine	[69]
	Biochanin A	[55]
	*Silybum marianum*	[56]
	Grape seed and skin extract	[63]
	EGCG	[49]
	Omega-3	[57]
	Flax seed oil	[53]
	Boerhavia diffusa	[73]
	*Malus domestica* L. Peel	[66]
	Phloretin	[45]
	Sorbus phnuashanesis	[62]
	Resveratrol	[72]
Decreased TR activity	Phloretin	[45]
Decreased GPx activity	*T. dioica* root	[68]
	*T. dioica* fruit	[67]
	Eugenol	[32]
	Taurine	[69]
	*Silybum marianum*	[56]
	Grape seed and skin extract	[63]
	EGCG	[49]
	Omega-3	[57]
	Flax seed oil	[53]
	*Malus domestica* L. Peel	[66]
	Phloretin	[45]
	Sorbus phnuashanesis	[62]
	Resveratrol	[72]
Increased GPx activity	Ellagic acid	[54]
Decreased GR activity	*T. dioica* root	[68]
	*T. dioica* fruit	[67]
	Taurine	[69]
	*Silybum marianum*	[56]
	*Malus domestica* L. Peel	[66]
Decreased GST activity	*T. dioica* root	[68]
	*T. dioica* fruit	[67]
	Eugenol	[32]
	Taurine	[69]
	*Silybum marianum*	[56]
	Omega-3	[57]
	Flax seed oil	[53]
Decreased G6PD activity	*Silybum marianum*	[56]
Downregulated HO-1	Naringin	[70]
	*Silybum marianum* (liver)	[56]
	Sorbus phnuashanesis	[62]
	Resveratrol	[33]
Elevated HO-1 expression	Imperatorin	[61]
	Sec-*O*-glucosylhamaudol ^2^	[61]
Elevated NQ01 expression	Imperatorin	[61]
	Sec-*O*-glucosylhamaudol	[61]
Decreased catalase activity	*T. dioica* root	[68]
	*T. dioica* fruit	[67]
	Taurine	[69]
	Biochanin A ^3^	[55]
	*Silybum marianum*	[56]
	Grape seed and skin extract	[63]
	Omega-3	[57]
	Flax seed oil	[53]
	Boerhavia diffusa	[73]
	*Malus domestica* L. Peel	[66]
	Sorbus phnuashanesis	[62]
	Resveratrol	[72]
Decreased GSH levels	Naringin	[70]
	*T. dioica* root	[68]
	*T. dioica* fruit	[67]
	Eugenol	[32]
	Resveratrol	[65]
	Taurine	[69]
	Biochanin A	[55]
	*Silybum marianum*	[56]
	Omega-3	[57]
	Flax seed oil	[53]
	Phloretin	[45]
No significant change in GSH	Ellagic acid	[54]
Short term GSH elevation followed by decrease	*Malus domestica* L. Peel	[66]
Increased GSSG	Selenium	[71]
	*T. dioica* root	[68]
	*T. dioica* fruit	[67]
Decreased GSH/GSSG ratio	Taurine	[69]
	Resveratrol	[33]
**ROS**		
Elevated H_2_O_2_	Grape seed and skin extract	[63]
Elevated mitochondrial ROS	Morphine	[60]
	EGCG	[49]
	*Malus domestica* L. Peel	[66]
	Phloretin	[45]
Elevated (H_2_O_2_, ONOO^−^, OH^−^)	Naringin	[70]
	Morphine	[60]
	Resveratrol	[65]
	Genistein	[65]
	Imperatorin	[61]
	Phloretin	[45]
	Sorbus phnuashanesis	[62]
	Boerhavia diffusa	[73]
	*Malus domestica* L. Peel	[66]
	Resveratrol	[33]
	Resveratrol	[72]
Lipid peroxidation (elevated MDA)	Naringin	[70]
	Eugenol	[32]
	Ellagic acid	[54]
	Biochanin A ^3^	[55]
	Grape seed and skin extract	[63]
	EGCG	[49]
	Taurine	[69]
	Omega-3	[57]
Increased 8-OHdG	α-lipoic acid	[59]
	Resveratrol	[33]
Elevated TBARS	*T. dioica* root	[68]
	*T. dioica* fruit	[67]
	*Silybum marianum*	[56]
	Flax seed oil	[53]
Increased XO	*Malus domestica* L. Peel	[66]
	Phloretin	[45]
Increased NOX activity (NOX2 and NOX4)	*Silybum marianum*	[56]
No change in NO	Resveratrol	[33]
Increased NO content	Naringin	[70]
**Apoptosis**		
LDH release	Taurine	[69]
	Imperatorin	[61]
	Sec-*O*-glucosylhamaudol	[61]
	EGCG	[49]
	Omega-3	[57]
	Boerhavia diffusa	[73]
	*Malus domestica* L. Peel	[66]
	Phloretin	[45]
	Sorbus phnuashanesis	[62]
	Resveratrol	[33]
	Resveratrol	[72]
Decreased cell viability	Morphine	[60]
	Resveratrol	[65]
	Genistein	[65]
	Taurine	[69]
	Imperatorin	[61]
	Sec-*O*-glucosylhamaudol	[61]
	EGCG	[49]
	Boerhavia diffusa	[73]
	*Malus domestica* L. Peel	[66]
	Phloretin	[45]
	Sorbus phnuashanesis	[62]
	Resveratrol	[72]
Apoptosis	Naringin	[70]
	Genistein	[64]
	Resveratrol + Genistein	[65]
	Taurine	[69]
	Imperatorin	[61]
	Sec-*O*-glucosylhamaudol	[61]
	EGCG	[49]
	Omega-3	[57]
	Boerhavia diffusa	[73]
	*Malus domestica* L. Peel	[66]
	Phloretin	[45]
	Sorbus phnuashanesis	[62]
	Resveratrol	[72]
DNA fragmentation	Morphine	[60]
	*T. dioica* root	[68]
	*T. dioica* fruit	[67]
	Genistein	[65]
	*Silybum marianum*	[56]
	Omega 3	[33]
	Resveratrol	[72]
Decreased cell growth via DNA synthesis	Morphine	[60]
Increased caspase-3 cleavage	Genistein	[65]
	EGCG	[49]
Elevated caspase-3 activity	Morphine	[60]
	Genistein	[64]
	Taurine	[69]
	Imperatorin ^3^	[61]
	Sec-*O*-glucosylhamaudol ^3^	[61]
	EGCG	[49]
	Sorbus phnuashanesis	[62]
	*Malus domestica* L. Peel	[66]
	Phloretin	[45]
	Resveratrol	[72]
Elevated caspase-8 activity	Sorbus phnuashanesis	[62]
Elevated caspase-9 activity	Sorbus phnuashanesis	[62]
Elevated cytochrome-c	Taurine	[69]
Proteolysis of PARP	Taurine	[69]
Increased BAX	Morphine	[60]
	Taurine	[69]
Increased Bad	Taurine	[69]
Increased PUMA	Morphine	[60]
Decreased Bcl_2_	Morphine	[60]
	Taurine	[69]
Decreased Bcl-xL	Morphine ^3^	[60]
	Taurine	[69]
Decreased Bcl2/BAX ratio	Sorbus phnuashanesis	[62]
Decreased CIAP1, CIAP2, XIAP	Morphine ^3^	[60]
Decreased Survivin	Morphine	[60]
Decreased P-Akt/Akt	Sorbus phnuashanesis	[62]
Upregulated TGF-β	Naringin	[70]
Increased SMAD3	Naringin	[70]
**Calcium and Membrane potential**		
Decreased NF-κB activity	Morphine	[60]
Phosphorylated NF-κB	Taurine	[69]
Calcium accumulation	Eugenol	[32]
	Genistein	[64]
	Taurine	[69]
	Imperatorin ^3^	[61]
	Sec-*O*-glucosylhamaudol ^3^	[61]
	Grape seed and skin extract	[63]
	EGCG	[49]
	Omega-3	[57]
	Boerhavia diffusa	[73]
	*Malus domestica* L. Peel	[66]
	Phloretin	[45]
	Resveratrol	[72]
	Resveratrol	[33]
Increased in Cav1.2	Genistein	[64]
Decreased Ca-ATPase activity	Eugenol	[32]
	*Silybum marianum*	[56]
	Phloretin	[45]
Decreased Na^+^/K^+^ ATPase activity	Naringin	[70]
	Eugenol	[32]
	*Silybum marianum*	[56]
Decreased transmembrane potential	Resveratrol	[65]
	Genistein	[65]
	Taurine	[69]
	Genistein	[64]
	Omega-3	[57]
	Phloretin	[45]
	Boerhavia diffusa	[73]
Elevated phosphorylation of JNK	Genistein	[64]
and p-38 MAPK	Taurine	[69]
**Mitochondria**		
Decreased activity at mito.	Naringin	[70]
complex I, III, IV	Phloretin	[45]
Decreased activity at mito.	Naringin	[70]
complex II	Morphine	[60]
Decreased ATP content	Phloretin	[45]
Decreased Mg^2+^ ATPase	*Silybum marianum*	[56]
Altered mitochondrial morphology	Naringin	[70]
	*Silybum marianum*	[56]
	Phloretin	[45]
Decreased OCR	Phloretin	[45]
Mito. swelling and pore opening	Phloretin	[45]
Decreased activities of heart mitochondrial enzymes	*Silybum marianum*	[56]
Decreased aconitase activity	Phloretin	[45]
Increased LC3-II/LC-31	Genistein ^2^	[65]
	Resveratrol ^2^	[65]

^1^ Partially restored; ^2^ further increased; ^3^ not restored.

## Supplementaly Materials

The following are available online at http://www.mdpi.com/2305-6304/5/4/38/s1. Table S1: Study Quality.

## References

[1] Mandal B.K., Suzuki K.T. (2002). Arsenic round the world: A review. Talanta.

[2] Chen C.J., Hsueh Y.M., Lai M.S., Shyu M.P., Chen S.Y., Wu M.M., Kuo T.L., Tai T.Y. (1995). Increased prevalence of hypertension and long-term arsenic exposure. Hypertension.

[3] Chen C.J., Chiou H.Y., Chiang M.H., Lin L.J., Tai T.Y. (1996). Dose-response relationship between ischemic heart disease mortality and long-term arsenic exposure. Arterioscler. Thromb. Vasc. Biol..

[4] Abhyankar L.N., Jones M.R., Guallar E., Navas-Acien A. (2012). Arsenic exposure and hypertension: A systematic review. Environ. Health Perspect..

[5] Tsuda T., Babazono A., Yamamoto E., Kurumatani N., Mino Y., Ogawa T., Kishi Y., Aoyama H. (1995). Ingested arsenic and internal cancer: A historical cohort study followed for 33 years. Am. J. Epidemiol..

[6] Wang F., Zhou X., Liu W., Sun X., Chen C., Hudson L.G., Liu K.J. (2013). Arsenite-induced ros/rns generation causes zinc loss and inhibits the activity of poly(adp-ribose) polymerase-1. Free Radic. Biol. Med..

[7] Wang X., Mandal A.K., Saito H., Pulliam J.F., Lee E.Y., Ke Z.J., Lu J., Ding S., Li L., Shelton B.J. (2012). Arsenic and chromium in drinking water promote tumorigenesis in a mouse colitis-associated colorectal cancer model and the potential mechanism is ros-mediated wnt/β-catenin signaling pathway. Toxicol. Appl. Pharmacol..

[8] Nasr R., Lallemand-Breitenbach V., Zhu J., Guillemin M.C., de Thé H. (2009). Therapy-induced pml/rara proteolysis and acute promyelocytic leukemia cure. Clin. Cancer Res..

[9] Zhang T., Lu H., Li W., Hu R., Chen Z. (2015). Identification of arsenic direct-binding proteins in acute promyelocytic leukaemia cells. Int. J. Mol. Sci..

[10] Lu J., Chew E.H., Holmgren A. (2007). Targeting thioredoxin reductase is a basis for cancer therapy by arsenic trioxide. Proc. Natl. Acad. Sci. USA.

[11] Arnér E.S., Holmgren A. (2000). Physiological functions of thioredoxin and thioredoxin reductase. Eur. J. Biochem..

[12] Lillig C.H., Holmgren A. (2007). Thioredoxin and related molecules—From biology to health and disease. Antioxid. Redox Signal..

[13] Powis G., Mustacich D., Coon A. (2000). The role of the redox protein thioredoxin in cell growth and cancer. Free Radic. Biol. Med..

[14] Urig S., Becker K. (2006). On the potential of thioredoxin reductase inhibitors for cancer therapy. Semin. Cancer Biol..

[15] Ducas R.A., Seftel M.D., Ducas J., Seifer C. (2011). Monomorphic ventricular tachycardia caused by arsenic trioxide therapy for acute promyelocytic leukaemia. J. R. Coll. Phys. Edinb..

[16] Mumford J.L., Wu K., Xia Y., Kwok R., Yang Z., Foster J., Sanders W.E. (2007). Chronic arsenic exposure and cardiac repolarization abnormalities with qt interval prolongation in a population-based study. Environ. Health Perspect..

[17] Vizzardi E., Zanini G., Antonioli E., D’Aloia A., Raddino R., Cas L.D. (2008). Qt prolongation: A case of arsenical pericardial and pleural effusion. Cardiovasc. Toxicol..

[18] Finsterer J., Ohnsorge P. (2013). Influence of mitochondrion-toxic agents on the cardiovascular system. Regul. Toxicol. Pharmacol..

[19] Wang X., Fang H., Huang Z., Shang W., Hou T., Cheng A., Cheng H. (2013). Imaging ros signaling in cells and animals. J. Mol. Med. (Berl.).

[20] Turrens J.F. (2003). Mitochondrial formation of reactive oxygen species. J. Physiol..

[21] Freinbichler W., Colivicchi M.A., Stefanini C., Bianchi L., Ballini C., Misini B., Weinberger P., Linert W., Varešlija D., Tipton K.F. (2011). Highly reactive oxygen species: Detection, formation, and possible functions. Cell. Mol. Life Sci..

[22] Moskovitz J., Yim M.B., Chock P.B. (2002). Free radicals and disease. Arch. Biochem. Biophys..

[23] Mandal P. (2017). Molecular insight of arsenic-induced carcinogenesis and its prevention. Naunyn-Schmiedeberg’s Arch. Pharmacol..

[24] Ajila C.M., Brar S.K., Verma M., Tyagi R.D., Godbout S., Valéro J.R. (2011). Extraction and analysis of polyphenols: Recent trends. Crit. Rev. Biotechnol..

[25] Vakifahmetoglu-Norberg H., Ouchida A.T., Norberg E. (2017). The role of mitochondria in metabolism and cell death. Biochem. Biophys. Res. Commun..

[26] Marín-García J., Goldenthal M.J. (2002). Understanding the impact of mitochondrial defects in cardiovascular disease: A review. J. Card. Fail..

[27] Peng T.I., Jou M.J. (2010). Oxidative stress caused by mitochondrial calcium overload. Ann. N. Y. Acad. Sci..

[28] Marín-García J., Goldenthal M.J. (2002). The mitochondrial organelle and the heart. Rev. Esp. Cardiol..

[29] Antico Arciuch V.G., Alippe Y., Carreras M.C., Poderoso J.J. (2009). Mitochondrial kinases in cell signaling: Facts and perspectives. Adv. Drug Deliv. Rev..

[30] Zhu X.D., Fan H.S., Zhao C.Y., Lu J., Ikoma T., Tanaka J., Zhang X.D. (2007). Competitive adsorption of bovine serum albumin and lysozyme on characterized calcium phosphates by polyacrylamide gel electrophoresis method. J. Mater. Sci. Mater. Med..

[31] Townsend D.M., Tew K.D., Tapiero H. (2003). The importance of glutathione in human disease. Biomed. Pharmacother..

[32] Binu P., Priya N., Abhilash S., Vineetha R.C., Nair R.H. (2017). Studies on curative efficacy of monoterpene eugenol on anti- leukemic drug arsenic trioxide induced cardiotoxicity. Biomed. Pharmacother..

[33] Zhang W., Guo C., Gao R., Ge M., Zhu Y., Zhang Z. (2013). The protective role of resveratrol against arsenic trioxide-induced cardiotoxicity. Evid. Based Complement. Alternat. Med..

[34] Mao W., Chen X., Yang T., Yin Y., Ge M., Luo M., Chen D., Qian X. (2012). A rapid fluorescent screening method for cellular sensitivity to anti-cancer compound. Cytotechnology.

[35] Quig D. (1998). Cysteine metabolism and metal toxicity. Altern. Med. Rev..

[36] He X., Ma Q. (2009). Induction of metallothionein i by arsenic via metal-activated transcription factor 1: Critical role of c-terminal cysteine residues in arsenic sensing. J. Biol. Chem..

[37] Hirano S., Kobayashi Y., Cui X., Kanno S., Hayakawa T., Shraim A. (2004). The accumulation and toxicity of methylated arsenicals in endothelial cells: Important roles of thiol compounds. Toxicol. Appl. Pharmacol..

[38] Li S., Zhao H., Wang Y., Shao Y., Wang B., Xing M. (2017). Regulation of autophagy factors by oxidative stress and cardiac enzymes imbalance during arsenic or/and copper induced cardiotoxicity in gallus gallus. Ecotoxicol. Environ. Saf..

[39] Banerjee P., Bhattacharyya S.S., Bhattacharjee N., Pathak S., Boujedaini N., Belon P., Khuda-Bukhsh A.R. (2009). Ascorbic acid combats arsenic-induced oxidative stress in mice liver. Ecotoxicol. Environ. Saf..

[40] Deneke S.M. (2000). Thiol-based antioxidants. Curr. Top. Cell. Regul..

[41] Zhao P., Guo Y., Zhang W., Chai H., Xing H., Xing M. (2017). Neurotoxicity induced by arsenic in gallus gallus: Regulation of oxidative stress and heat shock protein response. Chemosphere.

[42] Loboda A., Damulewicz M., Pyza E., Jozkowicz A., Dulak J. (2016). Role of nrf2/ho-1 system in development, oxidative stress response and diseases: An evolutionarily conserved mechanism. Cell. Mol. Life Sci..

[43] Kansanen E., Jyrkkänen H.K., Levonen A.L. (2012). Activation of stress signaling pathways by electrophilic oxidized and nitrated lipids. Free Radic. Biol. Med..

[44] Pace C., Banerjee T.D., Welch B., Khalili R., Dagda R.K., Angermann J. (2016). Monomethylarsonous acid, but not inorganic arsenic, is a mitochondria-specific toxicant in vascular smooth muscle cells. Toxicol. In Vitro Int. J. Publ. Assoc. BIBRA.

[45] Vineetha V.P., Soumya R.S., Raghu K.G. (2015). Phloretin ameliorates arsenic trioxide induced mitochondrial dysfunction in h9c2 cardiomyoblasts mediated via alterations in membrane permeability and etc complexes. Eur. J. Pharmacol..

[46] Kim J.Y., Choi J.Y., Lee H.J., Byun C.J., Park J.H., Cho H.S., Cho S.J., Jo S.A., Jo I. (2015). The green tea component (-)-epigallocatechin-3-gallate sensitizes primary endothelial cells to arsenite-induced apoptosis by decreasing c-jun n-terminal kinase-mediated catalase activity. PLoS ONE.

[47] Chen X., Shan H., Zhao J., Hong Y., Bai Y., Sun I., Pan Z., Zhang Y., Yang B., Du Z. (2010). L-type calcium current (ica,l) and inward rectifier potassium current (ik1) are involved in qt prolongation induced by arsenic trioxide in rat. Cell. Physiol. Biochem..

[48] Bashir S., Sharma Y., Irshad M., Gupta S.D., Dogra T.D. (2006). Arsenic-induced cell death in liver and brain of experimental rats. Basic Clin. Pharmacol. Toxicol..

[49] Sun T.L., Liu Z., Qi Z.J., Huang Y.P., Gao X.Q., Zhang Y.Y. (2016). (-)-Epigallocatechin-3-gallate (egcg) attenuates arsenic-induced cardiotoxicity in rats. Food Chem. Toxicol..

[50] Griesmacher A., Kindhauser M., Andert S.E., Schreiner W., Toma C., Knoebl P., Pietschmann P., Prager R., Schnack C., Schernthaner G. (1995). Enhanced serum levels of thiobarbituric-acid-reactive substances in diabetes mellitus. Am. J. Med..

[51] Li Q., Pogwizd S.M., Prabhu S.D., Zhou L. (2014). Inhibiting Na^+^/K^+^ atpase can impair mitochondrial energetics and induce abnormal Ca^2+^ cycling and automaticity in guinea pig cardiomyocytes. PLoS ONE.

[52] Mathews V.V., Paul M.V., Abhilash M., Manju A., Abhilash S., Nair R.H. (2013). Myocardial toxicity of acute promyelocytic leukaemia drug-arsenic trioxide. Eur. Rev. Med. Pharmacol. Sci..

[53] Varghese M.V., Abhilash M., Alex M., Sauganth Paul M.V., Prathapan A., Raghu K.G., Harikumaran Nair R. (2017). Attenuation of arsenic trioxide induced cardiotoxicity through flaxseed oil in experimental rats. Redox Rep..

[54] Hemmati A.A., Olapour S., Varzi H.N., Khodayar M.J., Dianat M., Mohammadian B., Yaghooti H. (2017). Ellagic acid protects against arsenic trioxide-induced cardiotoxicity in rat. Hum. Exp. Toxicol..

[55] Jalaludeen A.M., Lee W.Y., Kim J.H., Jeong H.Y., Ki K.S., Kwon E.G., Song H. (2015). Therapeutic efficacy of biochanin a against arsenic-induced renal and cardiac damage in rats. Environ. Toxicol. Pharmacol..

[56] Muthumani M., Prabu S.M. (2014). Silibinin potentially attenuates arsenic-induced oxidative stress mediated cardiotoxicity and dyslipidemia in rats. Cardiovasc. Toxicol..

[57] Varghese M.V., Abhilash M., Paul M.V., Alex M., Nair R.H. (2017). Omega-3 fatty acid protects against arsenic trioxide-induced cardiotoxicity in vitro and in vivo. Cardiovasc. Toxicol..

[58] Kumazaki M., Ando H., Kakei M., Ushijima K., Taniguchi Y., Yoshida M., Yamato S., Washino S., Koshimizu T.A., Fujimura A. (2013). Alpha-lipoic acid protects against arsenic trioxide-induced acute qt prolongation in anesthetized guinea pigs. Eur. J. Pharmacol..

[59] Kumazaki M., Ando H., Sasaki A., Koshimizu T.A., Ushijima K., Hosohata K., Oshima Y., Fujimura A. (2011). Protective effect of alpha-lipoic acid against arsenic trioxide-induced acute cardiac toxicity in rats. J. Pharmacol. Sci..

[60] Amini-Khoei H., Hosseini M.J., Momeny M., Rahimi-Balaei M., Amiri S., Haj-Mirzaian A., Khedri M., Jahanabadi S., Mohammadi-Asl A., Mehr S.E. (2016). Morphine attenuated the cytotoxicity induced by arsenic trioxide in h9c2 cardiomyocytes. Biol. Trace Elem. Res..

[61] Hu L., Sun J., Li H., Wang L., Wei Y., Wang Y., Zhu Y., Huo H., Tan Y. (2016). Differential mechanistic investigation of protective effects from imperatorin and sec-o-glucosylhamaudol against arsenic trioxide-induced cytotoxicity in vitro. Toxicol In Vitro.

[62] Yu X., Wang Z., Shu Z., Li Z., Ning Y., Yun K., Bai H., Liu R., Liu W. (2017). Effect and mechanism of sorbus pohuashanensis (hante) hedl. Flavonoids protect against arsenic trioxide-induced cardiotoxicity. Biomed. Pharmacother..

[63] Sfaxi I., Charradi K., Limam F., El May M.V., Aouani E. (2015). Grape seed and skin extract protects against arsenic trioxide induced oxidative stress in rat heart. Can. J. Physiol. Pharmacol..

[64] Fan Y., Wang C., Zhang Y., Hang P., Liu Y., Pan Z., Wang N., Du Z. (2013). Genistein ameliorates adverse cardiac effects induced by arsenic trioxide through preventing cardiomyocytes apoptosis. Cell. Physiol. Biochem..

[65] Fan Y., Chen M., Meng J., Yu L., Tu Y., Wan L., Fang K., Zhu W. (2014). Arsenic trioxide and resveratrol show synergistic anti-leukemia activity and neutralized cardiotoxicity. PLoS ONE.

[66] Vineetha V.P., Girija S., Soumya R.S., Raghu K.G. (2014). Polyphenol-rich apple (*Malus domestica* L.) peel extract attenuates arsenic trioxide induced cardiotoxicity in h9c2 cells via its antioxidant activity. Food Funct..

[67] Bhattacharya S., Das S.K., Haldar P.K. (2014). Arsenic induced myocardial toxicity in rats: Alleviative effect of trichosanthes dioica fruit. J. Diet. Suppl..

[68] Bhattacharya S., Haldar P.K. (2013). Trichosanthes dioica root alleviates arsenic induced myocardial toxicity in rats. J. Environ. Pathol. Toxicol. Oncol..

[69] Ghosh J., Das J., Manna P., Sil P.C. (2009). Taurine prevents arsenic-induced cardiac oxidative stress and apoptotic damage: Role of nf-kappa b, p38 and jnk mapk pathway. Toxicol. Appl. Pharmacol..

[70] Adil M., Kandhare A.D., Ghosh P., Bodhankar S.L. (2016). Sodium arsenite-induced myocardial bruise in rats: Ameliorative effect of naringin via tgf-β/smad and nrf/ho pathways. Chem. Biol. Interact..

[71] Krohn R.M., Lemaire M., Negro Silva L.F., Lemarié C., Bolt A., Mann K.K., Smits J.E. (2016). High-selenium lentil diet protects against arsenic-induced atherosclerosis in a mouse model. J. Nutr. Biochem..

[72] Zhao X.Y., Li G.Y., Liu Y., Chai L.M., Chen J.X., Zhang Y., Du Z.M., Lu Y.J., Yang B.F. (2008). Resveratrol protects against arsenic trioxide-induced cardiotoxicity in vitro and in vivo. Br. J. Pharmacol..

[73] Vineetha V.P., Prathapan A., Soumya R.S., Raghu K.G. (2013). Arsenic trioxide toxicity in h9c2 myoblasts—Damage to cell organelles and possible amelioration with boerhavia diffusa. Cardiovasc. Toxicol..

[74] Zaccolo M. (2009). Camp signal transduction in the heart: Understanding spatial control for the development of novel therapeutic strategies. Br. J. Pharmacol..

[75] Kurian G.A., Paddikkala J. (2010). Role of mitochondrial enzymes and sarcoplasmic atpase in cardioprotection mediated by aqueous extract of *Desmodium gangeticum* (L.) dc root on ischemic reperfusion injury. Indian J. Pharm. Sci..

[76] Liu D., Duan X., Dong D., Bai C., Li X., Sun G., Li B. (2013). Activation of the nrf2 pathway by inorganic arsenic in human hepatocytes and the role of transcriptional repressor bach1. Oxidative Med. Cell. Longev..

[77] Pi J., Qu W., Reece J.M., Kumagai Y., Waalkes M.P. (2003). Transcription factor nrf2 activation by inorganic arsenic in cultured keratinocytes: Involvement of hydrogen peroxide. Exp. Cell. Res..

[78] Wang X.J., Sun Z., Chen W., Eblin K.E., Gandolfi J.A., Zhang D.D. (2007). Nrf2 protects human bladder urothelial cells from arsenite and monomethylarsonous acid toxicity. Toxicol. Appl. Pharmacol..

[79] Meng D., Wang X., Chang Q., Hitron A., Zhang Z., Xu M., Chen G., Luo J., Jiang B., Fang J. (2010). Arsenic promotes angiogenesis in vitro via a heme oxygenase-1-dependent mechanism. Toxicol. Appl. Pharmacol..

[80] Abiko Y., Shinkai Y., Sumi D., Kumagai Y. (2010). Reduction of arsenic-induced cytotoxicity through nrf2/ho-1 signaling in hepg2 cells. J. Toxicol. Sci..

[81] Sumi D., Abe K., Himeno S. (2013). Arsenite retards the cardiac differentiation of rat cardiac myoblast h9c2 cells. Biochem. Biophys. Res. Commun..

[82] Kao Y.H., Yu C.L., Chang L.W., Yu H.S. (2003). Low concentrations of arsenic induce vascular endothelial growth factor and nitric oxide release and stimulate angiogenesis in vitro. Chem. Res. Toxicol..

[83] Lawrence T. (2009). The nuclear factor nf-kappab pathway in inflammation. Cold Spring Harb. Perspect. Biol..

[84] Lee P.C., Ho I.C., Lee T.C. (2005). Oxidative stress mediates sodium arsenite-induced expression of heme oxygenase-1, monocyte chemoattractant protein-1, and interleukin-6 in vascular smooth muscle cells. Toxicol. Sci..

[85] Martin-Pardillos A., Sosa C., Sorribas V. (2013). Arsenic increases pi-mediated vascular calcification and induces premature senescence in vascular smooth muscle cells. Toxicol. Sci..

[86] Lim K.M., Shin Y.S., Kang S., Noh J.Y., Kim K., Chung S.M., Yun Y.P., Chung J.H. (2011). Potentiation of vasoconstriction and pressor response by low concentration of monomethylarsonous acid (mma(iii)). Toxicol. Lett..

[87] Calatayud M., Devesa V., Vélez D. (2013). Differential toxicity and gene expression in caco-2 cells exposed to arsenic species. Toxicol. Lett..

[88] Scholz C., Wieder T., Stärck L., Essmann F., Schulze-Osthoff K., Dörken B., Daniel P.T. (2005). Arsenic trioxide triggers a regulated form of caspase-independent necrotic cell death via the mitochondrial death pathway. Oncogene.

[89] Correa F., Buelna-Chontal M., Hernández-Reséndiz S., García-Niño W.R., Roldán F.J., Soto V., Silva-Palacios A., Amador A., Pedraza-Chaverrí J., Tapia E. (2013). Curcumin maintains cardiac and mitochondrial function in chronic kidney disease. Free Radic. Biol. Med..

[90] Tanida I., Ueno T., Kominami E. (2008). Lc3 and autophagy. Autophagosome Phagosome.

[91] Han Y.H., Kim S.Z., Kim S.H., Park W.H. (2008). Arsenic trioxide inhibits the growth of calu-6 cells via inducing a g2 arrest of the cell cycle and apoptosis accompanied with the depletion of gsh. Cancer Lett..

[92] Partridge M.A., Huang S.X., Hernandez-Rosa E., Davidson M.M., Hei T.K. (2007). Arsenic induced mitochondrial dna damage and altered mitochondrial oxidative function: Implications for genotoxic mechanisms in mammalian cells. Cancer Res..

[93] Manach C., Scalbert A., Morand C., Rémésy C., Jiménez L. (2004). Polyphenols: Food sources and bioavailability. Am. J. Clin. Nutr..

[94] Cassady J.M., Zennie T.M., Chae Y.H., Ferin M.A., Portuondo N.E., Baird W.M. (1988). Use of a mammalian cell culture benzo(a)pyrene metabolism assay for the detection of potential anticarcinogens from natural products: Inhibition of metabolism by biochanin a, an isoflavone from *Trifolium pratense* L. Cancer Res..

[95] Hyson D.A. (2011). A comprehensive review of apples and apple components and their relationship to human health. Adv. Nutr. Int. Rev. J..

[96] Abe L.T., Lajolo F.M., Genovese M.I. (2012). Potential dietary sources of ellagic acid and other antioxidants among fruits consumed in brazil: Jabuticaba (myrciaria jaboticaba (vell.) berg). J. Sci. Food Agric..

[97] Kannan M.M., Quine S.D., Sangeetha T. (2012). Protective efficacy of ellagic acid on glycoproteins, hematological parameters, biochemical changes, and electrolytes in myocardial infarcted rats. J. Biochem. Mol. Toxicol..

[98] Devika P.T., Stanely Mainzen Prince P. (2008). (-)Epigallocatechingallate protects the mitochondria against the deleterious effects of lipids, calcium and adenosine triphosphate in isoproterenol induced myocardial infarcted male wistar rats. J. Appl. Toxicol..

[99] Devika P.T., Stanely Mainzen Prince P. (2008). (-)Epigallocatechin-gallate (egcg) prevents mitochondrial damage in isoproterenol-induced cardiac toxicity in albino wistar rats: A transmission electron microscopic and in vitro study. Pharmacol. Res..

[100] Nègre-Salvayre A., Salvayre R. (1992). Quercetin prevents the cytotoxicity of oxidized ldl on lymphoid cell lines. Free Radic. Biol. Med..

[101] Rossi L., Lippe G., Marchese E., De Martino A., Mavelli I., Rotilio G., Ciriolo M.R. (1998). Decrease of cytochrome c oxidase protein in heart mitochondria of copper-deficient rats. Biometals.

[102] Lee J., Giordano S., Zhang J. (2012). Autophagy, mitochondria and oxidative stress: Cross-talk and redox signalling. Biochem. J..

[103] Muthumani M., Prabu S.M. (2012). Silibinin potentially protects arsenic-induced oxidative hepatic dysfunction in rats. Toxicol. Mech. Methods.

[104] Negi A.S., Kumar J.K., Luqman S., Shanker K., Gupta M.M., Khanuja S.P. (2008). Recent advances in plant hepatoprotectives: A chemical and biological profile of some important leads. Med. Res. Rev..

[105] Roden D.M. (2008). Cellular basis of drug-induced torsades de pointes. Br. J. Pharmacol..

[106] Berquin I.M., Edwards I.J., Chen Y.Q. (2008). Multi-targeted therapy of cancer by omega-3 fatty acids. Cancer Lett..

[107] Gülçin I., Beydemir S., Alici H.A., Elmastaş M., Büyükokuroğlu M.E. (2004). In vitro antioxidant properties of morphine. Pharmacol. Res..

[108] Rosenblum A., Marsch L.A., Joseph H., Portenoy R.K. (2008). Opioids and the treatment of chronic pain: Controversies, current status, and future directions. Exp. Clin. Psychopharmacol..

[109] Costa-Malaquias A., Almeida M.B., Monteiro J.R.S., de Matos Macchi B., do Nascimento J.L.M., Crespo-Lopez M.E. (2014). Morphine protects against methylmercury intoxication: A role for opioid receptors in oxidative stress?. PLoS ONE.

[110] Kanesaki T., Saeki M., Ooi Y., Suematsu M., Matsumoto K., Sakuda M., Saito K., Maeda S. (1999). Morphine prevents peroxynitrite-induced death of human neuroblastoma sh-sy5y cells through a direct scavenging action. Eur. J. Pharmacol..

[111] Lee J., Kim M.S., Park C., Jung E.B., Choi D.H., Kim T.Y., Moon S.K., Park R. (2004). Morphine prevents glutamate-induced death of primary rat neonatal astrocytes through modulation of intracellular redox. Immunopharmacol. Immunotoxicol..

[112] Qian L., Tan K.S., Wei S.J., Wu H.M., Xu Z., Wilson B., Lu R.B., Hong J.S., Flood P.M. (2007). Microglia-mediated neurotoxicity is inhibited by morphine through an opioid receptor-independent reduction of nadph oxidase activity. J. Immunol..

[113] He H., Huh J., Wang H., Kang Y., Lou J., Xu Z. (2016). Mitochondrial events responsible for morphine’s cardioprotection against ischemia/reperfusion injury. Toxicol. Appl. Pharmacol..

[114] Calder P.C. (2012). Mechanisms of action of (n-3) fatty acids. J. Nutr..

[115] Stanley W.C., Khairallah R.J., Dabkowski E.R. (2012). Update on lipids and mitochondrial function: Impact of dietary n-3 polyunsaturated fatty acids. Curr. Opin. Clin. Nutr. Metab. Care.

[116] Jong C.J., Azuma J., Schaffer S. (2012). Mechanism underlying the antioxidant activity of taurine: Prevention of mitochondrial oxidant production. Amino Acids.

[117] Xu Y.J., Saini H.K., Zhang M., Elimban V., Dhalla N.S. (2006). Mapk activation and apoptotic alterations in hearts subjected to calcium paradox are attenuated by taurine. Cardiovasc. Res..

[118] Sole M.J., Jeejeebhoy K.N. (2000). Conditioned nutritional requirements and the pathogenesis and treatment of myocardial failure. Curr. Opin. Clin. Nutr. Metab. Care.

[119] Calabrese E.J., Baldwin L.A. (2003). Inorganics and hormesis. Crit. Rev. Toxicol..

[120] Brglez Mojzer E., Knez Hrnčič M., Škerget M., Knez Ž., Bren U. (2016). Polyphenols: Extraction methods, antioxidative action, bioavailability and anticarcinogenic effects. Molecules.

[121] Espinosa-Diez C., Miguel V., Mennerich D., Kietzmann T., Sánchez-Pérez P., Cadenas S., Lamas S. (2015). Antioxidant responses and cellular adjustments to oxidative stress. Redox Biol..

[122] Manach C., Williamson G., Morand C., Scalbert A., Rémésy C. (2005). Bioavailability and bioefficacy of polyphenols in humans. I. Review of 97 bioavailability studies. Am. J. Clin. Nutr..

[123] Croft K.D. (2016). Dietary polyphenols: Antioxidants or not?. Arch. Biochem. Biophys..

[124] Gebel T.W. (2001). Genotoxicity of arsenical compounds. Int. J. Hyg. Environ. Health.

[125] Wang C.H., Jeng J.S., Yip P.K., Chen C.L., Hsu L.I., Hsueh Y.M., Chiou H.Y., Wu M.M., Chen C.J. (2002). Biological gradient between long-term arsenic exposure and carotid atherosclerosis. Circulation.

[126] Rahman M., Tondel M., Ahmad S.A., Chowdhury I.A., Faruquee M.H., Axelson O. (1999). Hypertension and arsenic exposure in bangladesh. Hypertension.

[127] Verret W.J., Chen Y., Ahmed A., Islam T., Parvez F., Kibriya M.G., Graziano J.H., Ahsan H. (2005). A randomized, double-blind placebo-controlled trial evaluating the effects of vitamin E and selenium on arsenic-induced skin lesions in bangladesh. J. Occup. Environ. Med..

[128] Mahata J., Argos M., Verret W., Kibriya M.G., Santella R.M., Ahsan H. (2008). Effect of selenium and vitamin E supplementation on plasma protein carbonyl levels in patients with arsenic-related skin lesions. Nutr. Cancer.

[129] Kibriya M.G., Jasmine F., Argos M., Verret W.J., Rakibuz-Zaman M., Ahmed A., Parvez F., Ahsan H. (2007). Changes in gene expression profiles in response to selenium supplementation among individuals with arsenic-induced pre-malignant skin lesions. Toxicol. Lett..

[130] Krohn R.M., Raqib R., Akhtar E., Vandenberg A., Smits J.E. (2016). A high-selenium lentil dietary intervention in bangladesh to counteract arsenic toxicity: Study protocol for a randomized controlled trial. Trials.

[131] Tenore G.C., Caruso D., Buonomo G., D’Avino M., Campiglia P., Marinelli L., Novellino E. (2017). A healthy balance of plasma cholesterol by a novel annurca apple-based nutraceutical formulation: Results of a randomized trial. J. Med. Food.

[132] Cai Y., Zhang J., Chen N.G., Shi Z., Qiu J., He C., Chen M. (2017). Recent advances in anticancer activities and drug delivery systems of tannins. Med. Res. Rev..

[133] Shafiei S.S., Solati-Hashjin M., Samadikuchaksaraei A., Kalantarinejad R., Asadi-Eydivand M., Abu Osman N.A. (2015). Epigallocatechin gallate/layered double hydroxide nanohybrids: Preparation, characterization, and in vitro anti-tumor study. PLoS ONE.

[134] Adlercreutz H. (2007). Lignans and human health. Crit. Rev. Clin. Lab. Sci..

[135] Sanna V., Siddiqui I.A., Sechi M., Mukhtar H. (2013). Resveratrol-loaded nanoparticles based on poly(epsilon-caprolactone) and poly(d,l-lactic-co-glycolic acid)-poly(ethylene glycol) blend for prostate cancer treatment. Mol. Pharm..

[136] Carletto B., Berton J., Ferreira T.N., Dalmolin L.F., Paludo K.S., Mainardes R.M., Farago P.V., Favero G.M. (2016). Resveratrol-loaded nanocapsules inhibit murine melanoma tumor growth. Colloids Surf. B Biointerfaces.

[137] Shao J., Li X., Lu X., Jiang C., Hu Y., Li Q., You Y., Fu Z. (2009). Enhanced growth inhibition effect of resveratrol incorporated into biodegradable nanoparticles against glioma cells is mediated by the induction of intracellular reactive oxygen species levels. Colloids Surf. B Biointerfaces.

[138] Mohan A., Narayanan S., Sethuraman S., Krishnan U.M. (2014). Novel resveratrol and 5-fluorouracil coencapsulated in pegylated nanoliposomes improve chemotherapeutic efficacy of combination against head and neck squamous cell carcinoma. Biomed. Res. Int..

[139] Guo W., Li A., Jia Z., Yuan Y., Dai H., Li H. (2013). Transferrin modified peg-pla-resveratrol conjugates: In vitro and in vivo studies for glioma. Eur. J. Pharmacol..

[140] Sassi N., Mattarei A., Azzolini M., Bernardi P., Szabo I., Paradisi C., Zoratti M., Biasutto L. (2014). Mitochondria-targeted resveratrol derivatives act as cytotoxic pro-oxidants. Curr. Pharm. Des..

[141] Pham J., Brownlow B., Elbayoumi T. (2013). Mitochondria-specific pro-apoptotic activity of genistein lipidic nanocarriers. Mol. Pharm..

[142] Richter C.K., Skulas-Ray A.C., Fleming J.A., Link C.J., Mukherjea R., Krul E.S., Kris-Etherton P.M. (2017). Effects of isoflavone-containing soya protein on ex vivo cholesterol efflux, vascular function and blood markers of cvd risk in adults with moderately elevated blood pressure: A dose-response randomised controlled trial. Br. J. Nutr..

[143] Kelso G.F., Porteous C.M., Coulter C.V., Hughes G., Porteous W.K., Ledgerwood E.C., Smith R.A., Murphy M.P. (2001). Selective targeting of a redox-active ubiquinone to mitochondria within cells: Antioxidant and antiapoptotic properties. J. Biol. Chem..

[144] Rao V.A., Klein S.R., Bonar S.J., Zielonka J., Mizuno N., Dickey J.S., Keller P.W., Joseph J., Kalyanaraman B., Shacter E. (2010). The antioxidant transcription factor nrf2 negatively regulates autophagy and growth arrest induced by the anticancer redox agent mitoquinone. J. Biol. Chem..

[145] Chandran K., Aggarwal D., Migrino R.Q., Joseph J., McAllister D., Konorev E.A., Antholine W.E., Zielonka J., Srinivasan S., Avadhani N.G. (2009). Doxorubicin inactivates myocardial cytochrome c oxidase in rats: Cardioprotection by mito-q. Biophys. J..

