# Supplementary material for: Antioxidants Protect against Arsenic Induced Mitochondrial Cardio-Toxicity
Source: Toxics. 2017 Dec 5;5(4):38. doi: 10.3390/toxics5040038 (PMC5750566; doi:10.3390/toxics5040038)
Supplement: Supplementary file 1 [file toxics-05-00038-s001.pdf]

Supplementary Materials: Antioxidants Protect against Arsenic Induced Mitochondrial Cardio-Toxicity

Clare Pace, Ruben Dagda and Jeff Angermann

Table S1. Study Quality

	Criterion	Yes (n)	No (n)	Yes %	Average (SD)
1.	Was the study purpose clearly stated?	25	0	100%	-
2.	Number of technical replicates (*in vitro*)	-	-	-	5.8 (1.6)
3.	Number of endpoints assayed	-	-	-	14.6 (9.6)
4.	Were source and type of cells/animals listed?	23	2	92%	
5.	What is the number of experimental groups?	-	-	-	5.8 (2.2)
6.	Did study specify the number of animals per group? (*in vivo*)	24	1	96%	6.7 (1.6)
7.	Was gender of animals specified? (*in vivo*)	25	0	100%	-
8.	Were animals randomly assigned to experimental groups?	13	6	68.4%	-
9.	Was a power analysis performed?	0	24	0%	-
	Was variability assessed:				
10.	Between subjects?	25	0	100%	-
	Within subjects?	0	24	0%	-
11.	Was statistical method fully reported?	22	3	88%	-
12.	Was statistical method appropriate?	20	2	91.3%	-
13.	Was numerical data reported?	18	7	72%	-
14.	Were effect sizes reported?	0	25	0%	-
	What statistics were reported for primary outcomes?				
15.	Mean(SE)	11	14	44%	-
	Mean(SEM)	14	11	56%	-
16.	Was funding source reported?	17	8	68%	-
17.	Average number of citations/article	-	-	-	12.1 (20.5)
18.	H Index	-	-	-	86.8 (50.8)
